# Salinity and diatom host community jointly shape chytrid parasite diversity along a natural salinity gradient

**DOI:** 10.1093/ismeco/ycag269

**Published:** 2026-09-25

**Authors:** Jonna Kangas, Simon Bilik, Lumi Haraguchi, Kaisa Kraft, Maliheh Mehrshad, Conny Sjöqvist, Silke Van den Wyngaert

**Affiliations:** Department of Biology, University of Turku, Turku 20014, Finland; Institute for Research and Applications of Fuzzy Modeling, University of Ostrava, Ostrava 70200, Czech Republic; Research Infrastructure, Finnish Environmental Institute, Helsinki 00790, Finland; Research Infrastructure, Finnish Environmental Institute, Helsinki 00790, Finland; Department of Aquatic Sciences and Assessment, Science for Life Laboratory, Swedish University of Agricultural Sciences, Uppsala 750 07, Sweden; Department of Environmental and Marine Biology, Åbo Akademi University, Turku 20500, Finland; Department of Biology, University of Turku, Turku 20014, Finland

**Keywords:** salinity, chytrid parasites, phytoplankton bloom, diversity, Baltic Sea, Illumina, IFCB

## Abstract

Parasitic fungi of the phylum Chytridiomycota are known parasites of phytoplankton, yet little is known about drivers of their diversity and host interaction dynamics in marine and brackish habitats. This study investigates the diversity and distribution patterns of phytoplankton-chytrid associations during a phytoplankton spring bloom along a natural salinity gradient of the Baltic Sea at a bi-weekly temporal resolution. Direct linking of chytrids to their host species using single-cell picking and sequencing (SCPS), combined with community composition analyses using amplicon sequencing and quantitative imaging flow cytometry, enabled a high-resolution profiling of spatiotemporal patterns of host–chytrid interactions and infection dynamics along the salinity gradient. This showed an increase in the relative abundance of parasitic chytrids coupled with the increase in phytoplankton biomass towards the end of the spring bloom. Moreover, SCPS revealed 5 novel chytrid-phytoplankton associations and aligning their sequences with spatiotemporal amplicon sequencing data showed a greater diversity and relative abundance of parasitic chytrids below a salinity of 8. Above this threshold, chytrid diversity and abundance declined considerably, coinciding with host community turnover and a shift towards marine non-fungal parasites. These results indicate that salinity structures chytrid communities primarily through its effects on host composition and parasite competition rather than through direct physiological limitation alone. Our findings leverage a combination of methods to provide novel insights at single-cell resolution on drivers of parasitic chytrids associated with phytoplankton blooms. Covering a spatial salinity gradient and temporal dynamics, our data highlights the role of phytoplankton blooms in shaping fungal communities.

## Graphical abstract

**Graphical abstract:**
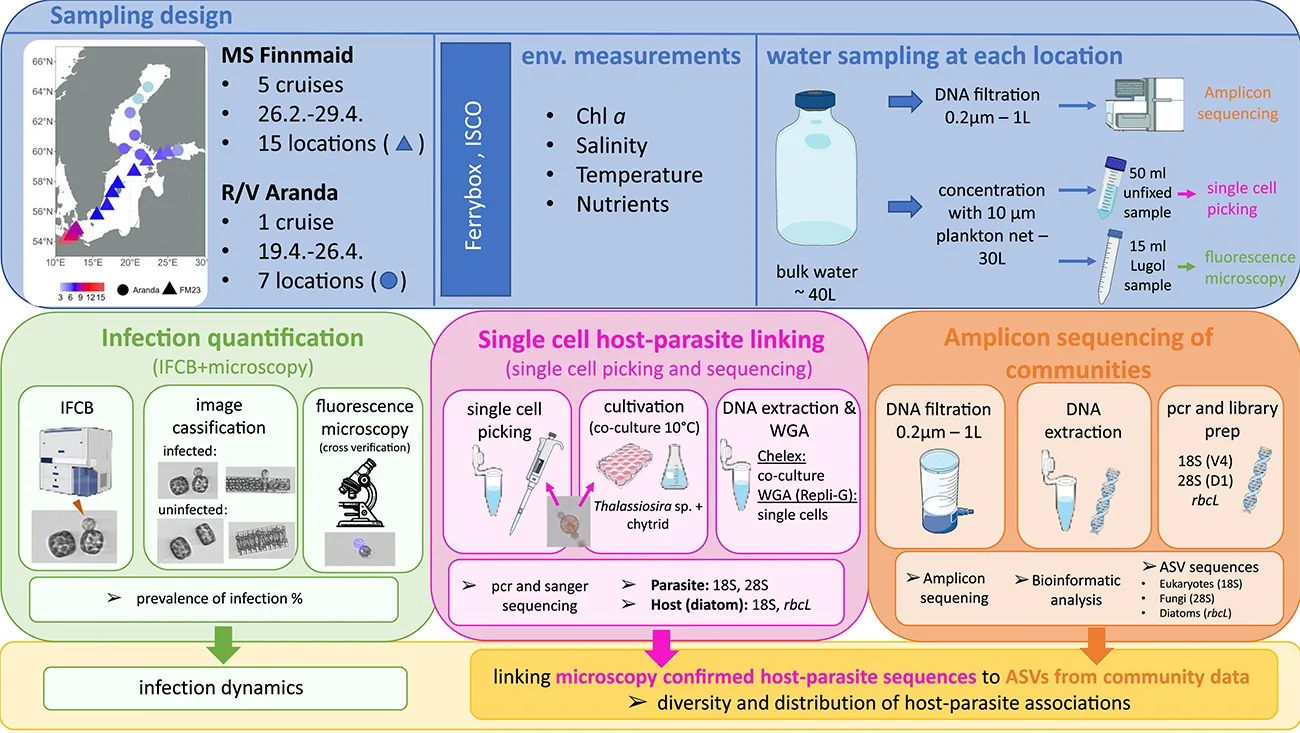

## Introduction

Parasitism can promote stability and resilience at the ecosystem level through its impact on biodiversity and food web interactions [1]. At the base of aquatic food webs, parasitic interactions may trigger cascading effects, altering food web dynamics and ecosystem functioning [2].

Fungal parasites of the phylum Chytridiomycota, often referred to as chytrids, infect all major phytoplankton taxa and are frequently found to act as extensive infective agents during phytoplankton blooms in freshwater lakes [3–5]. Most prominently studied systems include the freshwater diatom *Asterionella formosa* infected by the parasitic chytrids *Rhizophydium planktonicum* and *Zygophlyctis asterionellae* (formerly *Zygorhizidium planktonicum*) and the filamentous cyanobacteria genus *Planktothrix* infected by *Rhizophidium megarrhizum* [3, 5, 6]. Chytrid epidemics can have significant ecological implications, potentially altering phytoplankton succession and diversity [7], including the genetic diversity of phytoplankton populations [8]. Moreover, chytrid zoospores serve as a nutritious food source for zooplankton [9, 10], forming significant trophic links in aquatic food webs [11] and channeling carbon transfer to higher trophic levels [12].

Amplicon-based studies show that chytrids are also prevalent among marine and brackish fungal plankton [13–16], and parasitic chytrids have recently been reported in coastal summer phytoplankton blooms in the northern Baltic Sea at salinities between 3 and 7 [17–19]. However, little is known about the diversity, distribution, and dynamics of phytoplankton chytrid interactions in marine and brackish systems and across salinity gradients.

Salinity is a significant environmental factor influencing species distributions and diversity across multiple taxa, including phytoplankton and fungi [16, 20–22]. Several studies have shown that chytrid distribution varies with salinity, with chytrids often dominating the fungal community in brackish or freshwater conditions [13, 16, 23, 24]. Nevertheless, there are studies that found chytrids to dominate marine fungal communities of the Mediterranean Sea, where relative abundance of chytrids was strongly coupled with the blooms of specific dinoflagellates *Alexandrium minutum* and *Ostreopsis* sp. [25, 26]. These observations indicate that physicochemical drivers may not be the only determinant of chytrid distribution, as host or substrate specificity and abundance can impose additional constraints on chytrid dynamics [27]. This is further complicated by the fact that chytrids represent different lifestyles ranging from saprotrophs to obligate parasites interacting with a diverse range of hosts and substrates [27, 28]. This functional diversity combined with the scarcity of reference sequences for brackish and marine parasitic chytrids, limits our ability to infer their ecological roles from amplicon-based surveys alone [29].

Furthermore, studies conducted at broader spatial scales often have limited temporal resolution and do not specifically resolve or identify phytoplankton–chytrid parasite interactions, which are typically highly dynamic during phytoplankton bloom events [30]. Thus, previous studies assessing chytrid diversity patterns across salinity gradients [13, 16, 23, 24] have not been able to fully disentangle host–parasite associations and effects of salinity on chytrid-host dynamics.

To bypass these limitations, we used a novel integrated approach where we combined amplicon sequencing with single-cell picking and sequencing (SCPS) of individual host–parasite pairs in order to generate reference sequences with known parasitic lifestyle, and an *in situ* automated imaging FlowCytobot (IFCB) for high-throughput analysis of chytrid–host interactions and spatiotemporal distribution patterns. This allowed us to resolve coupled host-chytrid spatiotemporal dynamics with an unprecedented resolution.

The Baltic Sea provides an ideal system for reliable interpretation of both abiotic (salinity) and biotic (host) impacts on chytrid diversity and distribution. It is characterized by a stable and broad horizontal salinity gradient [31], and phytoplankton diversity is strongly shaped by salinity where communities are at a near-stable state at local salinity conditions [20]. Here, we applied our integrated methodological approach combining amplicon sequencing, SCPS, and IFCB on samples collected along a latitudinal salinity gradient in the Baltic Sea ranging from salinities 3 and 16 over 5 sampling trips covering the spring bloom period. The high-resolution dataset generated in this study allowed us to resolve how salinity influenced the spatial distribution of chytrid parasites and further disentangle the impact of host prevalence on their distribution and host-chytrid dynamics.

## Materials and methods

### Study area and sample collection

A schematic overview of the sampling design and analytical workflow (infection quantification, amplicon sequencing, and single-cell host–parasite linking through SCPS) is provided in Fig. S1. Samples were collected on board the ship-of-opportunity M/S Finnmaid during 5 cruises, conducted every 2 weeks between February and May 2023. The shipping route follows a 1132 km transect between Helsinki (Finland) and Travemünde (Germany). The ship is equipped with a FerryBox [32], which is maintained by the Finnish Environment Institute (Syke). An automated system pumps water at a nominal depth of 5 m every 100–200 m and records chlorophyll *a* (Chl *a*) fluorescence, salinity, and temperature. Fifteen sampling locations were selected, covering a salinity gradient from 5 to 16 (Fig. 1). Phosphate, silicate, nitrite, and nitrate were collected with an automated water sampler (ISCO Inc., Nebraska, USA) and analyzed as in [33, 34]. Bulk water (approx. 40 L) was manually collected from a tap. One liter was filled in autoclaved Nalgene bottles for DNA filtration. Approximately 30 L of water was prefiltered through a 250 μm mesh to remove mesozooplankton and concentrated to 200 ml using a 10 μm mesh plankton net (KC Denmark, Denmark). A 50 ml subsample was stored at 4°C on board and used for the isolation of infected phytoplankton cells or colonies. Another 15 ml was fixed with neutral Lugol’s solution (final concentration 1%) for fluorescence microscopy analysis (Invitrogen EVOS M7000, Thermo Fisher Scientific, USA).

**Figure 1:**
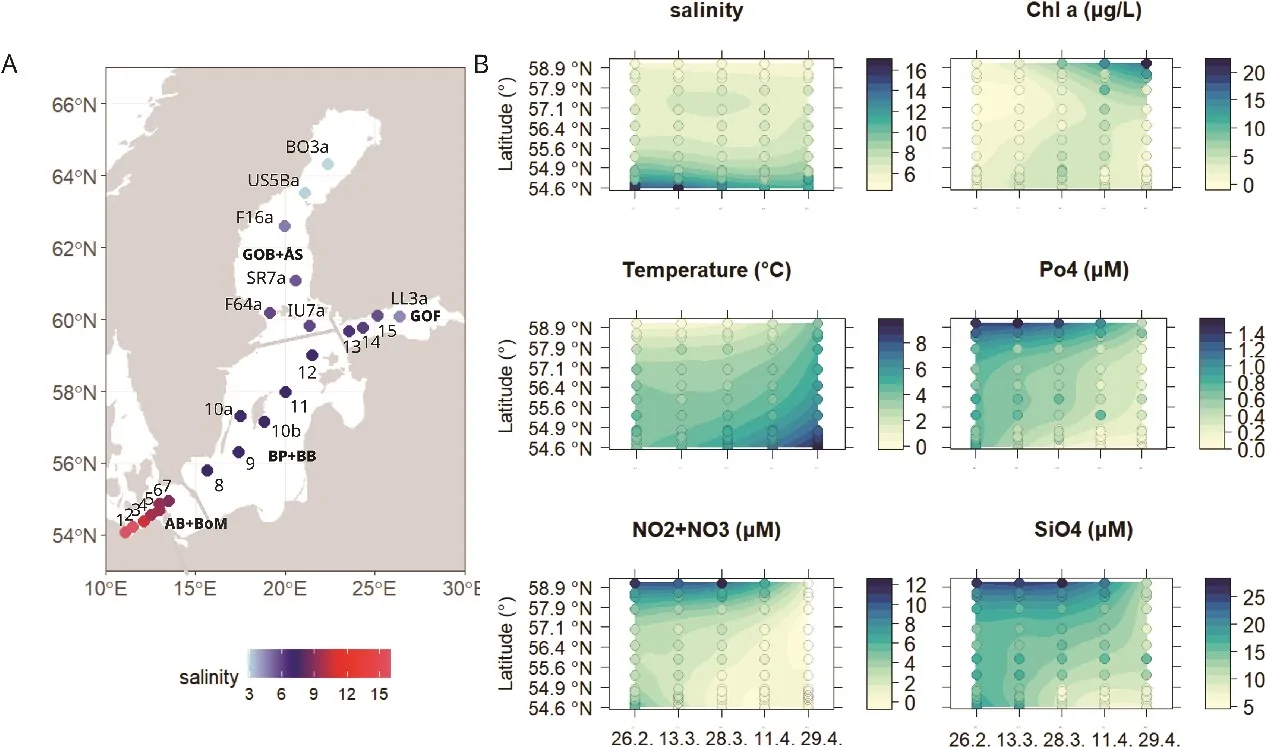
(A) Map of the sampling locations, (B) environmental conditions along the repeated Finnmaid sampling transect between GOF and AB+BoM (locations 15-1). The frequency of sampling locations was higher in the southern part of the transect to cover the steep salinity gradient in this area (approx. 8–16). The sampling points are color coded by their corresponding salinities from light blue (salinity 3) to red (salinity 16).

Seven additional samples were collected as described above at sampling sites from the Gulf of Bothnia and Åland Sea. Samples were collected on board of R/V Aranda (Syke) during the 19th–26th of April 2023 (Fig. S1). Details on exact sampling dates, locations, and environmental parameters measured for both sampling transects are provided in Supplementary Data 1.

### Quantification of phytoplankton and parasite infections

An IFCB [35] was coupled to the FerryBox system on board of M/S Finnmaid, processing a ~5 ml aliquot every ~23 min along the transect. The IFCB operated on a chlorophyll *a* trigger to target photosynthetic organisms in a size range of ~5 to ~150 μm (see more detailed description in [36, 37]). The IFCB images were classified into taxonomic groups using a Convolutional Neural Network-based classifier [38] with some modifications (Supplementary Methods).

Images of infected taxa (Fig. S2) were first categorized into anomalous (putative infections) and non-anomalous (healthy cells) using an anomaly detection algorithm [39]. Target taxa included *Pauliella taeniata*, Centrales (*Thalassiosira* spp. and *Coscinodiscus* spp.), and *Guinardia delicatula*. These preliminary classifications were then manually verified and classified as infected or non-infected following [40] (Supplementary Methods). Prevalence of infection was calculated for each infected host species as follows: *nr. infected images/(nr. uninfected images + nr. infected images)*. Fluorescence microscopy (Invitrogen EVOS M7000, Thermo Fisher Scientific, USA) on concentrated Lugol-fixed samples was done to confirm and quantify chytrid infections [41] (details in Supplementary Methods).

### Single-cell picking and cultivation, whole genome amplification, and sequencing

Individual infected phytoplankton cells or colonies were isolated from concentrated unfixed samples. Cells were picked with a micropipette under an inverted light microscope (Olympus CK40, Japan) and washed three times in artificial seawater-based sterile growth medium f/2 + Si [42]. After washing, single cells were individually transferred into 1.5 ml low-bind Eppendorf tubes and stored at −80°C until further processing. To establish host–parasite co-cultures, infected cells were transferred to a culture of *Thalassiosira* sp. isolated in 2022 from the Baltic Proper (59.66° N, 23.45° E) and incubated at 10°C under a 16:8 h light:dark cycle.

DNA of the infected cells was extracted and amplified by multiple displacement amplification (MDA) using the Qiagen REPLI-g Single Cell Kit (Qiagen, Germantown, Maryland, USA), according to manufacturer’s instructions, following modifications from [43]. MDA products were diluted by 1:100. DNA of established co-cultures was extracted by adding 80 μL 5% Chelex solution (Bio Rad, California, USA) to a pelleted cell suspension. Cells were heated at 95°C for 20 min. DNA concentrations were measured with the Qubit fluorometer.

The 18S and the 28S region of the parasite was amplified using fungal specific forward and reverse primers NS1/NS4 [44] and LROR/LR5 [45, 46]. The 18S region and the chloroplast encoded *rbcL* marker gene were amplified using diatom-specific primers SSU-F1289/SSU-R1772 [47] and rbcL-F/rbcLR3-DEG [48, 49]. DNA regions were amplified with the My Taq HS red mix (Meridian Bioscience, Memphis, Tennessee, USA). PCR cycling conditions for each primer pair are described in the Supplementary Methods. PCR products were sequenced with the Sanger method by Macrogen Europe. Sequences were edited, quality checked, and aligned with BioEdit [50].

### DNA extraction and amplicon sequencing of fungal and phytoplankton communities

A 0.5 L water sample was filtered on a 0.2 μm polycarbonated track etch membrane filter (Cytiva, Malborough, MA, USA), and filters were stored in −20°C on board and later in −80°C in the lab until further analysis. Genomic DNA was extracted with the Qiagen DNeasy PowerSoil kit according to manufacturer’s instructions. PCR, library preparation, and sequencing (2 × 300 bp) were performed by LGC Genomics (Berlin, Germany) on an Illumina Miseq platform.

To target the eukaryotic community, the V4 region of the 18S was sequenced using the forward and reverse primers V4_Balzano_F `5-CCA GCA SCY GCG GTA ATT CC-3' and V4_Balzano_R `5-ACTTTCGTTCTTGATYRR-3' [51], respectively. For targeting chytrids and the broader fungal community, the D1 region of the 28S was sequenced using the forward and reverse primers ITS4ngsF `5-GCATATCAATAAGCGSAGGA-3' and LF402R `5-TTCCCTTTYARCAATTTCAC-3' [30], respectively. To target the diatom community, the chloroplast encoded *rbcL* marker gene was sequenced using the forward and reverse primers Diat_rbcL_708F `5-AGG TGA AGT TAA AGG TTC ATA CTT DAA-3' and R3_1 `5-CCT TCT AAT TTA CCA ACA ACT G-3' [49], respectively.

### Bioinformatic analysis

Raw sequence data of 18S (eukaryotes), 28S (fungi), and *rbcL* (diatoms) amplicon sequences were adapter clipped using cutadapt (v. 4.6). Sequence data was further preprocessed using the nf-core ampliseq pipeline (v. 2.8.0) [52], including the following steps: primers were removed with cutadapt (v. 3.4), and reads were trimmed and quality filtered with DADA2 (v. 1.2.8) [53]. The 18S ASVs (Amplicon Sequence Variants) were taxonomically annotated against the PR2 database (v.5.0.0) [54] with the nf-core ampliseq pipeline as described in GitHub (https://github.com/nf-core/ampliseq/blob/master/docs/output.md). The 28S ASVs were annotated against the SILVA (v. 138.1) database [55] and the *rbcL* ASVs against the R-syst::diatom database (v. 9) [56] using blastn (v.2.15.0) with an Evalue of 1e-5. The 18S data was cleared of metazoans and plantae, the 28S data cleared of non-fungal ASVs, and the *rbcL* data cleared of non-diatom ASVs using the filter function from the dplyr package (v. 1.2.1) in R (v. 4.2.1). In the fungal 28S dataset, sequences with <85% identity at the phylum level were filtered out [57]. After filtration, read abundances of 18S, 28S, and *rbcL* datasets were transformed to relative abundances using the decostand function with the method set to “total” from the vegan package (v. 2.7.3) [58].

Sanger sequences of the infected diatom host (*rbcL*) and the parasite (28S), obtained from host–parasite single-cell pairs and cultures, were aligned to the respective *rbcL* and 28S amplicon datasets using blastn (v.2.15.0). This step links microscopy-confirmed host–parasite pairs to specific ASVs detected in the environmental amplicon dataset, enabling us to track the distribution and relative abundance of these interacting species across samples. An ASV was considered identical to a diatom host or chytrid parasite isolate when it had a minimum sequence identity threshold of 99% and a minimum query length of 200 bps. This subset of host and parasite ASVs was used in subsequent statistical analyses. The 18S and 28S amplicon datasets were queried against the ParAquaSeq database [29] to identify other known zoosporic parasites of phytoplankton.

### Phylogenetic analysis

A phylogenetic analysis was done with the 28S and the *rbcL* sanger sequences obtained from cultures and single-cell isolates to clarify the phylogenetic position of parasite (28S) and host (*rbcL*). Sequences were aligned with MAFFT (v7.475), manually edited with BioEdit and trimmed with trimAl (v. 1.2rev59). A maximum likelihood (ML) tree was inferred with IQTree (v. 1.6.12) with the best fit model (JC + G4) chosen based on the corrected Akaike information criterion (AIC), and branch fit values were assessed with standard nonparametric bootstrap analysis (1000 replicates).

### Statistical analyses

Statistical analyses and data visualization were done with R (v. 4.2.1) using R packages vegan (v. 2.7.3) [58] and ggplot2 (v. 4.0.3) [59]. Alpha and beta diversity of the diatom host (*rbcL*) and chytrid parasite community (28S) was analyzed. ASV richness, Shannon and Simpson diversity were calculated with the diversity function, and beta diversity as Bray–Curtis dissimilarity was calculated with the vegdist function.

To identify spatial patterns of diversity, sampling locations were categorized into four sub-basins based on their location and corresponding salinity ranges as follows: Gulf of Bothnia and Åland Sea (GOB + ÅS) (salinity 3–5), Gulf of Finland (GOF) (salinity 5–6), Baltic Proper and Bornholm Basin (BP + BB) (salinity 7–8), Arkona Basin and Bay of Mecklenburg (AB+BoM) (salinity 8–16) (Table S1). The sub-basins were tested for significant differences in diatom and chytrid beta diversity with permutational multivariate analysis of variance (PERMANOVA) using the adonis2 function. Betadisper was used to test for homogeneity in dispersion between the sub-basins. An ordination with principal coordinates analysis (PCoA) was performed with cmdscale to visualize beta diversity of diatom host and chytrid parasite communities across the sub-basins.

To identify drivers of chytrid parasite diversity, the unique effects of salinity, host alpha, and beta diversity were tested on the parasitic chytrid community composition. This was done with a partial distance-based redundancy analysis using the capscale function while controlling for the effects of sampling trip and temperature with the condition term. To account for multicollinearity, highly intercorrelated variables (>0.7) were left out of the model (Fig. S3). Variance partitioning with varpart was done to measure the unique and shared contributions of salinity, host diversity, and control variables. A correlation analysis with Spearman’s rank correlation using base R functions was done to test for relationships between the relative abundances of parasite and host ASVs, as well as parasite ASVs and environmental variables.

## Results

### Environmental conditions and phytoplankton bloom development varied across the salinity gradient

Salinity ranged from 5.16 in GOF to 16.27 in AB+BoM (Fig. 1B). At T1 (26th of February), temperature ranged from 0.46°C in GOF to 4.31°C in AB+BoM and increased by T5 (29th of April) to 4.03°C in GOF and 9.24°C in AB+BoM. In GOB + ÅS (19th–26th of April), temperature ranged between 0.04°C and 3.33°C. Depletion of inorganic nutrients was coupled with the spring bloom development, shown by the spatiotemporal dynamics of Chl *a*, NO_2_ + NO_3_, PO_4_ and SiO_4_ along the sampling transect (Fig. 1B).

The timing of the bloom varied spatially along the sampling transect. Chl *a* concentration first peaked at the end of March in AB+BoM (7.6 μg L^−1^), followed by a peak mid of April in BP + BB (10.7 μg L^−1^), and a later peak at T5 in GOF, where concentrations reached up to 20.9 μg L^−1^ (Fig. 1B). Phytoplankton biomass (μg L^−1^) showed a similar pattern, reaching highest concentrations in GOF at the end of April (T5) (Fig. 2A). In GOB + ÅS, Chl *a* ranged between 0.9 μg L^−1^ (BO3a) and 9.51 μg L^−1^ (SR7a) (Supplementary Data 1).

**Figure 2:**
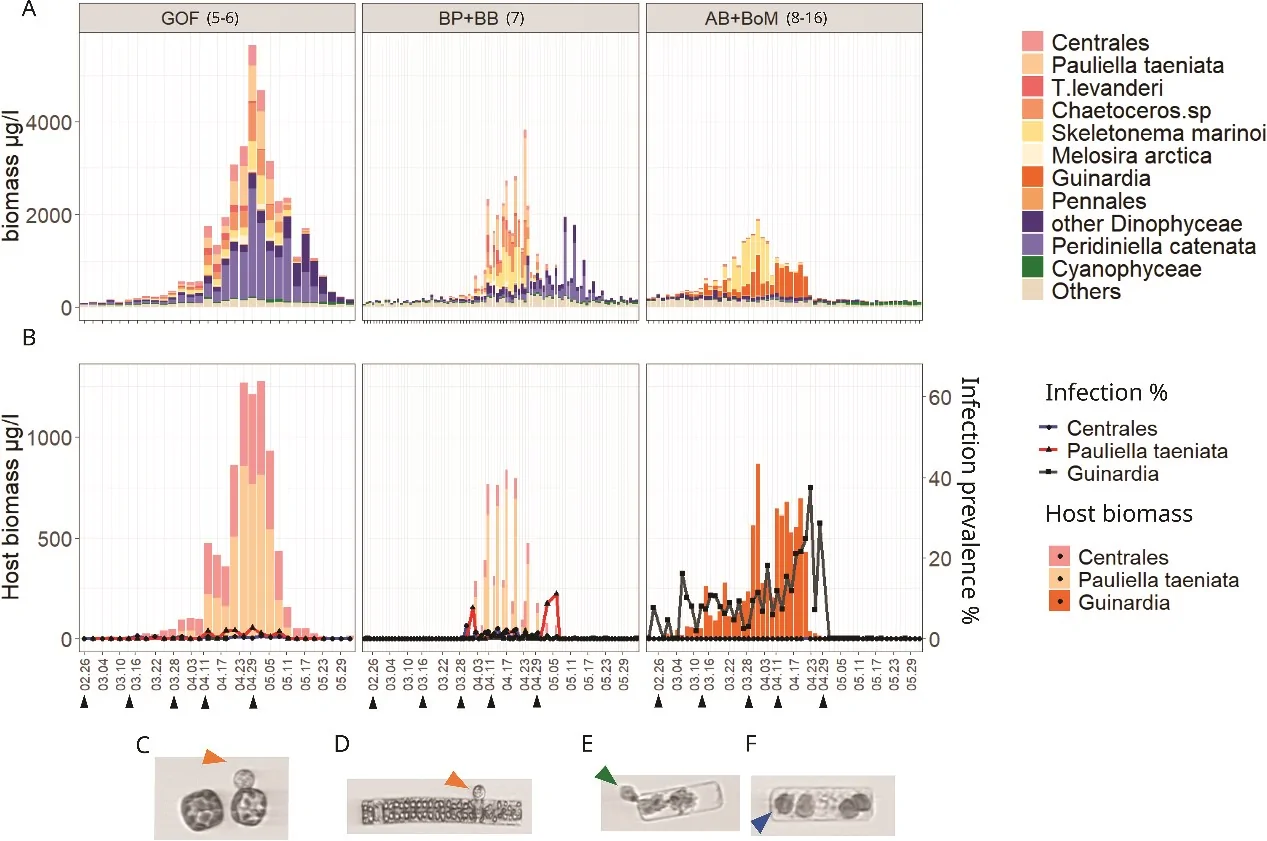
Temporal development of (A) spring bloom phytoplankton community composition (total biomass μg L^−1^) and (B) total host population biomass and prevalence of infection in each sub-basin. (C) Infected Centrales, (D) infected *P. taeniata*, (E) and (F) infected *G. delicatula*. Orange arrows indicate putative chytrid infections, green arrow putative *Prisonia* sp. infection, and blue arrow putative *Cryothecomonas* sp. infection. The latitudinal data are categorized into three main sub-basins: GOF = Gulf of Finland, BP + BB=Baltic Proper and Bornholm Basin; AB+BoM = Arkona Basin and Bay of Mecklenburg. Black arrowheads indicate the dates of the five sampling trips.

### Infection dynamics differed between sub-basins

Phytoplankton community and infection dynamics differed across sub-basins along the salinity gradient (Fig. 2A and B). In GOF, the community was dominated by diatoms (*Chaetoceros* sp., Centrales, *P. taeniata,* and dinoflagellates. Chytrid infections were observed on *P. taeniata* and Centrales with maximum infection prevalences of 2.7% and 0.7%, respectively. In BP + BB, the diatoms *Skeletonema* sp., *Chaetoceros* sp., and *P. taeniata* initially dominated the spring bloom, followed by a shift toward dinoflagellate dominance. Here, chytrid infections were observed on *P. taeniata* and Centrales (Fig. 2C and D), with maximum infection prevalences of 11.1% and 3.2%, respectively. In AB+BoM, the community was dominated by *Skeletonema* and *G. delicatula*. *G. delicatula* was heavily infected by the parasitic nanoflagellates *Pirsonia* sp. and *Cryothecomonas* sp. (Fig. 2E and F), reaching a combined infection prevalence of 37%. No chytrid infections were detected by IFCB in AB+BoM.

Microscopy confirmed infections on Centrales in GOF and on *P. taeniata* and Centrales in BP + BB. In AB+BoM microscopy revealed chytrid infections on *Thalassiosira* sp., with infection prevalences of 1.8% and 2.8% at locations 1 and 7, respectively (Fig. S4).

### Single-cell picking and sequencing of phytoplankton-chytrid associations revealed novel diatom-chytrid associations at both ends of the salinity gradient

Good quality sequences were obtained from 14 single infected phytoplankton cells/colonies and 2 cultures (Table 1). Parasite 28S and 18S sequences revealed five distinct chytrid taxa and one parasitic nanoflagellate (Figs 3 and 4). The *rbcL* sequences confirmed infected hosts to be *P. taeniata*, *G. delicatula*, and two distinct *Thalassiosira* species, one isolated from the northern sub-basins GOB and BP + BB, and the other from the southern AB+BoM (Fig. S5A). These are hereafter referred to as *Thalassiosira* sp.1 (northern) and *Thalassiosira* sp.2 (southern).

**Table 1:** Table of isolated parasites and their hosts.

**Isolate ID**	**Isolation type**	**Host**	**Parasite taxonomy**	**Location and year**	**Salinity**
31-*Thalassiosira* sp. 1 35-*Thalassiosira* sp. 1. S12-*Thalassiosira* sp. 1	single cell single cell culture	*Thalassiosira* sp. 1	Rhizophydiales sp. 1	lat 58.67 N lon 20.51 E 2023	7.08
SR5-*Thalassiosira* sp. 1	culture	*Thalassiosira* sp. 1	Rhizophydiales sp. 2	lat 61.05 N lon 19.35 E 2022	5.6
SR7-*Thalassiosira* sp.1	single cell	*Thalassiosira* sp. 1	Rhizophydiales sp. 3	lat 61.08 N lon 20.60 E 2023	5.5
5-*Thalassiosira* sp. 2 6-*G. delicatula* 8-*Thalassiosira* sp. 2 16-*Thalassiosira* sp. 2 17-*Thalassiosira* sp 2.	single cell	*Thalassiosira* sp. 2 *G. delicatula*	Lobulomycetales sp. 1	lat 54.01 N lon 11.00 E 2023	16.3
6-*G. delicatula* 12-*G. delicatula* 20-*G. delicatula*	single cell	*G. delicatula*	*Pirsonia* sp.	lat 54.01 N lon 11.00 E 2023	16.3
4-*P. taeniata* 49-*P. taeniata* 51-*P. taeniata*	single cell	*P. taeniata*	Lobulomycetales sp. 2	lat 58.67 N lon 20.51 E 2023	7.08

**Figure 3:**
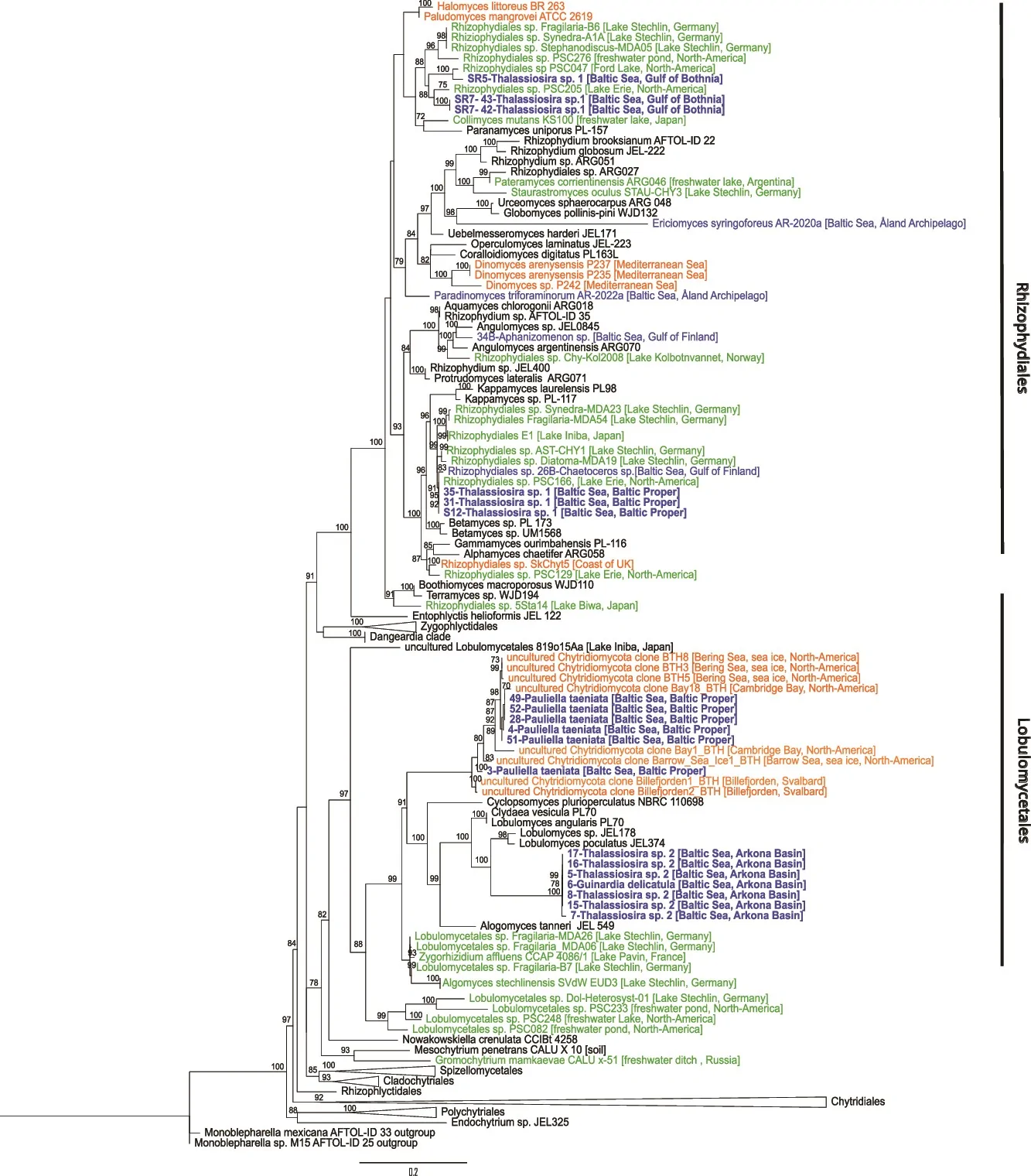
Maximum-likelihood phylogenetic tree of 28S rDNA sequences, including representatives from most orders within Chytridiomycota. Parasitic chytrids isolated in this study are marked in bold. Chytrids isolated from marine environments marked in orange, brackish environments in blue, and freshwater environments in green. Isolates marked in black originate from soil or other terrestrial habitats. Values in the nodes represent bootstrap (%) statistical support, with only values >70% shown.

**Figure 4:**
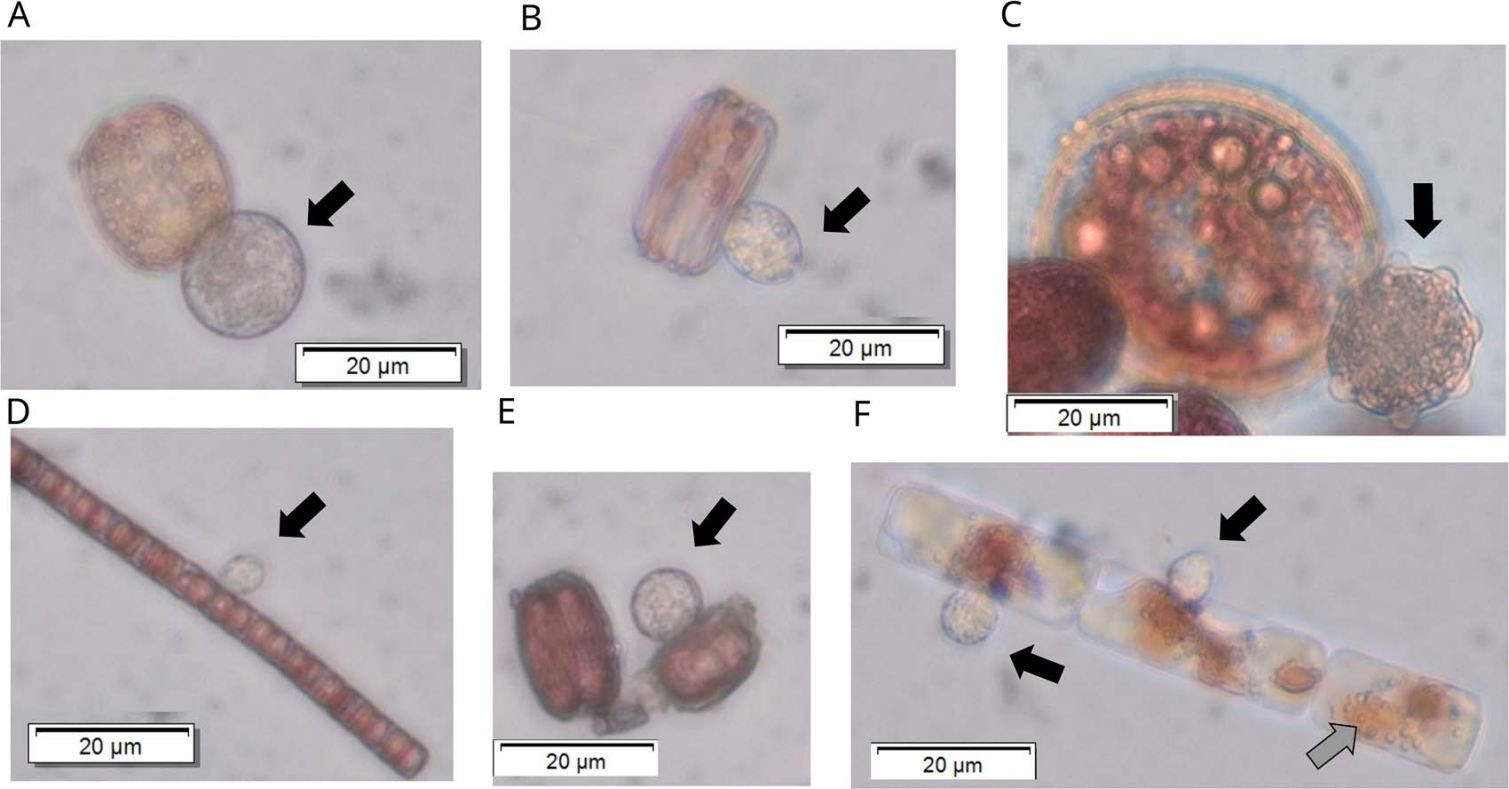
Light microscope images of the different isolated host–parasite species pairs: (A) *Thalassiosira* sp. 1 infected by Rhizophydiales sp. 1, (B) *Thalassiosira* sp. 1 infected by Rhizophydiales sp. 3, (C) *Thalassiosira* sp. 1 infected by Rhizophydiales sp. 2, (D) *P. taeniata* infected by Lobulomycetales sp. 2, (E) *Thalassiosira* sp. 2 infected by Lobulomycetales sp. 2, (F) *G. delicatula* infected simultaneously by both Lobulomycetales sp. 2 and *Pirsonia* sp. Black arrows point to mature chytrid sporangia attached to the host cell and the gray arrow points to *Pirsonia* sp. structure.

The chytrid infecting *P. taeniata* clustered with uncultured sequences from the Arctic Ocean [60] within the order Lobulomycetales (Fig. 3). *Thalassiosira* sp. 1 was infected by three chytrid lineages that clustered within the order Rhizophydiales. Two of these grouped near the family Halomycetacea. The third clustered with other diatom-associated parasites isolated from freshwater and brackish coastal Baltic Sea water samples (Rhizophydiales clade RH-1 sensu lato; [18, 60]) (Fig. 3) and showed 100% sequence similarity with a *Contricibra* sp. associated parasite previously isolated from Lake Erie [61]. *Thalassiosira* sp. 2 and *G. delicatula* were both infected by the same chytrid, which formed a distinct clade within the order Lobulomycetales. *G. delicatula* was also co-infected by a parasitic nanoflagellate clustering within the genus *Pirsonia* (Fig. S5B).

### Dynamics of fungal, chytrid, and parasitic chytrid communities were coupled with the spring bloom

The 18S dataset contained 1667 ASVs, of which 78 belonged to Fungi, and of these, 25 were assigned to Chytridiomycota (Fig. S6). The 28S dataset contained 2665 fungal ASVs, among them, 517 were assigned to Chytridiomycota (Fig. 5A). Of the 517 chytrid ASVs, 23 showed 99% similarity to parasitic chytrid sequences obtained from either single-cell or cultured isolates in this study (Supplementary Data 2). These 23 parasitic ASVs represented 4.4% of all chytrid ASVs and accounted for nearly half of all chytrid reads (42%) in the dataset (Fig. 5B).

**Figure 5:**
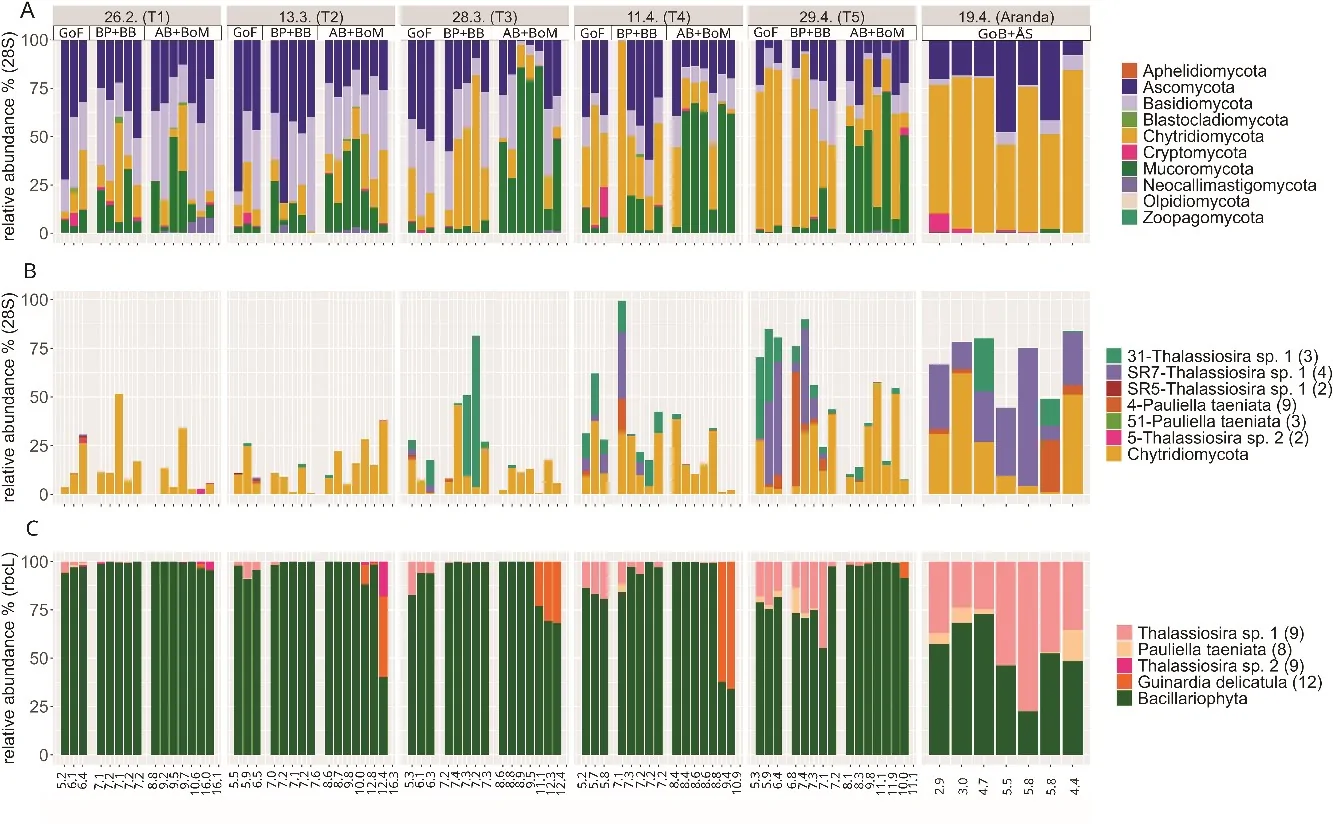
Relative abundance plots showing community composition along the salinity gradient during each sampling campaign. (A) Fungi (28S), (B) parasitic chytrids and Chytridiomycota and their proportion within the fungal community (28S), (C) diatom hosts and their proportion within Bacillariophyta (*rbcL*). The *x*-axis indicates the salinity at each sampling point. Brackets in legend title represent number of ASVs assigned to the taxa.

Ascomycota and Basidiomycota dominated fungal communities in most of the locations at T1 and T2 (Fig. 5A, Table S2). The relative abundance of Chytridiomycota first increased in BP + BB at T3, followed by an increase in GOF at T4. By T5, they dominated the fungal community in most of BP + BB and GOF (Fig. 5A). This successional increase was driven by an increasing proportion of parasitic chytrid ASVs in the fungal community, reaching 72% in BP + BB and 67% in GOF at T5 (Fig. 5B, Table S2). This pattern coincided with an increase in phytoplankton biomass, specific host biomass, and infection prevalence (Fig. 2), as well as an increase in the relative abundance of host species within the diatom community (Fig. 5C). In GoB + ÅS, Chytridiomycota dominated fungal communities with parasitic chytrids accounting for up to 71% of the fungal community (Fig. 5B, Table S2). In AB+BoM, the relative abundance of parasitic chytrid ASVs remained low (Fig. 5B, Table S2).

Parasitic chytrid ASVs corresponding to isolates from this study comprised the majority of the parasite community. Only a few rare ASVs, each never exceeding 1% of the fungal community, matched sequences of other parasitic chytrids. These included two marine species (*Dinomyces arenysensis* infecting dinoflagellates, SkChy5 infecting *Skeletonema* sp.) and three freshwater species (Zygophlyctidales sp., *Zygophlyctis planktonica, Protrudomyces lateralis*) (Fig. S5B).

### Diversity of parasitic chytrids and associated diatoms varied across the salinity gradient

Alpha diversity of both parasitic chytrids and ASVs identified as hosts of parasitic chytrids showed a significant negative relationship with salinity during the last two sampling trips (Fig. 6). Similar relationships between ASV richness and salinity were found for fungal, Chytridiomycota, and diatom communities (Fig. S7). At the peak and end of the phytoplankton bloom (T4, T5, Aranda), parasitic chytrid and their associated host communities were separated by a salinity threshold of 8 (Fig. 7). Both parasitic chytrid communities (28S) and their host communities (*rbcL*) differed significantly between the sub-basins (ADONIS: pseudo-*F* = 3.68; df = 3; R2 = 0.173; *P* = .001, pseudo-*F* = 6.77; df = 3; R2 = 0.215; *P* = .001, respectively). *Post hoc* test with pairwise.adonis2 revealed that parasitic chytrid communities differed significantly between all sub-basins except between GOF and BP + BB, and host communities differed significantly between all sub-basins (Table S3). For host communities, dissimilarities resulted both due to heterogeneity in dispersion across groups (betadisper: F = 24.35, *P* = .001) and average community differences (Fig. 7B). For parasitic chytrid communities, heterogeneity in dispersion across groups was not evident (betadisper: F = 2.38, *P* = .093) (Table S4).

**Figure 6:**
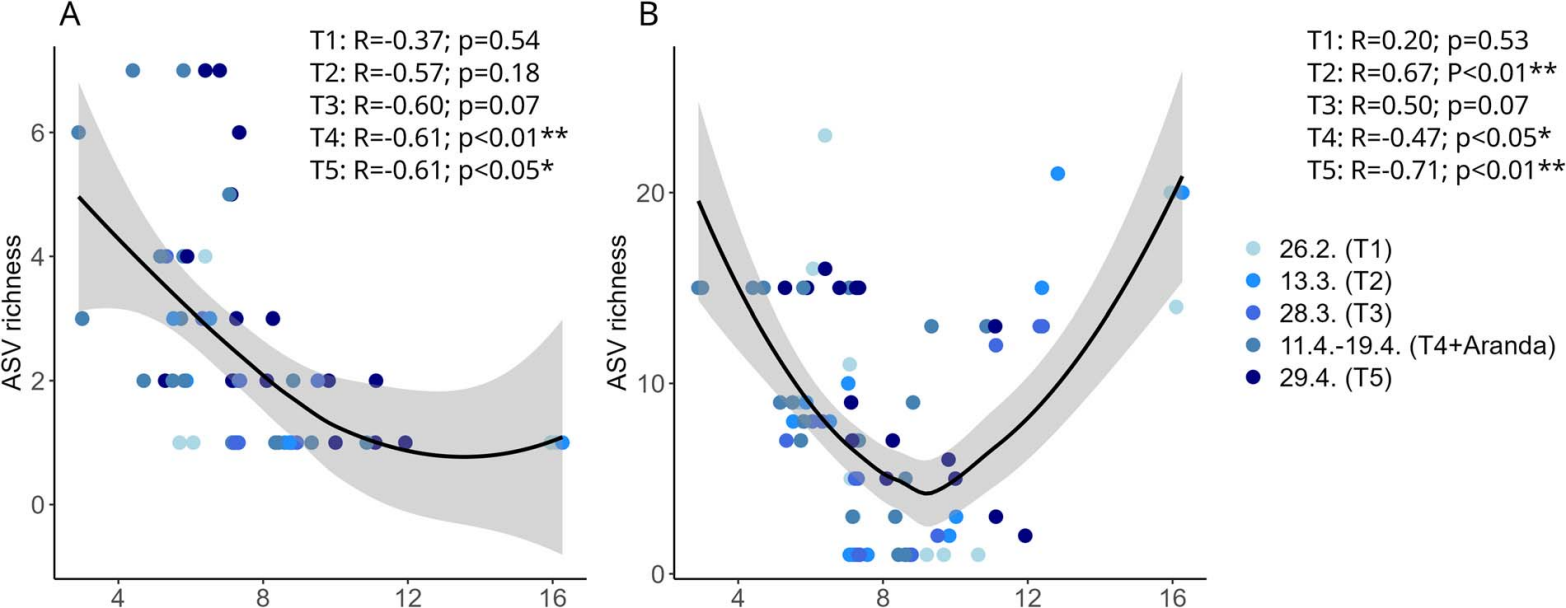
ASV richness of (A) chytrid parasite communities, (B) diatom host communities along the salinity gradient at each sampling trip. Pearson’s correlations describing the relationships of ASV richness and salinity are shown for each sampling trip individually.

**Figure 7:**
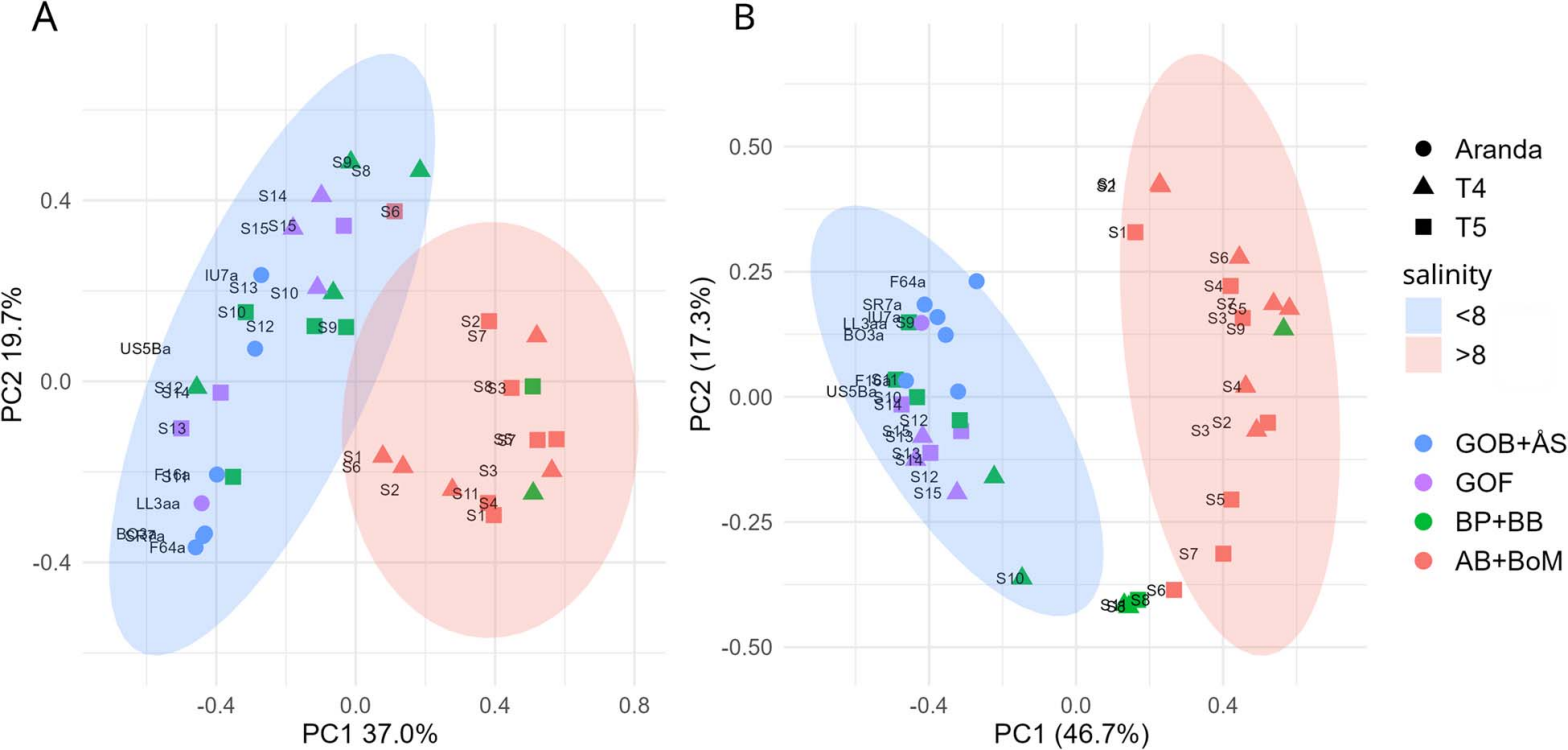
PCoA plot based on Bray–Curtis dissimilarities at sampling trips T4, T5, and Aranda. (A) Parasitic chytrid communities (28S) and (B) host communities (*rbcL*). Point colors represent the sub-basins and ellipses depict the salinity threshold at a salinity of 8 separating parasite and host communities.

The dbRDA revealed that parasitic chytrid community structure was significantly influenced by salinity (F = 2.20, *P* = .011) and host beta diversity (F = 1.95, *P* = .034) (Table S5, Fig. S8). According to variance partitioning, total variance explained by predictor variables salinity, host alpha and beta diversity, and control variables was 28%. Salinity and host diversity uniquely explained 2.1% and 3.0% of the variation in parasite community structure, respectively, while control variables explained 9.2% of the variation. Shared contributions of salinity and host diversity, salinity and control variables, and host diversity and control variables were 1.6%, 4.0%, and 8.2%, respectively. Residual variance accounted for 72% of variation (Table S6). Spearman’s correlation analysis showed that the relative abundances of parasite ASVs positively correlated with the relative abundances of their associated hosts (*r_s_* = 0.45, *P* = .001; *r_s_* = 0.76, *P* = .001; *r_s_* = 0.59, *P* = .001; *r_s_* = 0.21, *P* = .02; *r_s_* = 0.48, *P* = .001; *r_s_* = 0.30, *P* = .009) (Fig. S9). ASVs corresponding to chytrid parasites isolated from the northern parts of the Baltic Sea showed negative associations with salinity (*r_s_* = −0.37, *P* = .001; *r_s_* = −0.62, *P* = .001; *r_s_* = −0.41, *P* = .001; *r_s_* = −0.21, *P* = .07), whereas the ASVs of the chytrid parasite isolated from the southern part of the transect showed a weak positive association with salinity (*r_s_* = 0.22, *P* = .04) (Fig. S9).

## Discussion

Salinity is a significant driver of diversity in both macroscopic and microbial organisms [20, 62–64]. Diversity patterns along salinity gradients are often assessed at the community or single-taxon level, whereas biotic interactions are rarely accounted for, despite their recognized importance in shaping species distributions [65]. For obligate parasites, not only abiotic conditions but also host susceptibility, diversity, and abundance are critical for structuring parasite diversity [66]. Using the natural salinity gradient of the Baltic Sea, we investigated the drivers of host–parasite associations between chytrids and phytoplankton. Consistent with a study by Rojas-Jimenez and colleagues [16], we found that Chytridiomycota dominated fungal communities at salinities below 8 (Fig. 5A). Importantly, with our integrated methodological approach of single-cell host–parasite linking, community amplicon sequencing, and quantitative imaging flow cytometry, we move from descriptive community patterns to mechanistic insights and show that the dominance of Chytridiomycota at salinities <8 is driven by parasitic chytrids during the development of the phytoplankton spring bloom (Fig. 5B).

### Parasite and host diversity along the salinity gradient

Both the diversity and relative abundance of parasitic chytrids were higher at salinities below 8 and declined with increasing salinity (Figs 5B and 6A). Moreover, the pronounced decline around salinity 8 could suggest adaptation to lower saline brackish water conditions and a physiological barrier imposed by salinity. This pattern could be driven by more frequent freshwater inflow than marine intrusions into these brackish water areas [31], promoting locally adapted and more diverse communities characterized by freshwater lineages [67].

In line with this, we found that chytrids in the northern parts of the Baltic were closely related to freshwater [61] and Arctic lineages originating from the Bering Sea and Cambridge Bay of the western Arctic and sub-Arctic [60]. Similarly, their hosts *Thalassiosira* sp.1 and *P. taeniata* occur in freshwater, estuarine, and Arctic habitats such as the Arctic sea ice [68–70], implying that these chytrids might have colonized the Baltic Sea together with their hosts. Furthermore, some diatoms of the Baltic spring bloom, such as *P. taeniata*, are considered glacial relicts that colonized the Baltic Sea from Arctic waters after the last glacial period [69]. This would explain the close phylogenetic relationship between chytrids infecting *P. taeniata* and chytrids found in the Arctic (Fig. 3). The chytrid isolated from the southern Baltic, in turn, falls into a distinct lineage of the order Lobulomycetales, without close affiliation to known taxa (Fig. 3). This parasite and its hosts, *Thalassiosira* sp. 2 and *G. delicatula*, were restricted to the southern sampling locations (Figs 5C and 2), suggesting a different evolutionary trajectory with potentially different salinity tolerance and host specificity than that of the northern isolates. These findings point to a linked evolutionary history between chytrids and their diatom hosts in this study and are supported by their co-occurrence in both the molecular and quantitative data (Figs 2 and 5).

The similar trends in alpha and beta diversity of parasitic chytrids and their hosts along the salinity gradient (Figs. 6 & 7) suggest that the distribution of the preferred host species were also driving parasitic chytrids along the salinity gradient. Furthermore, as phytoplankton species composition shifted towards the dominance of the marine diatom *G. delicatula* at the southern end of the salinity gradient, the predominance of parasitic chytrids switched to predominance of the marine parasitic nanoflagellates *Pirsonia* sp. and *Cryothecomonas* sp. (Fig. 2), which were found to infect *G. delicatula* in marine environments [71, 72]. Thus, differing host composition and salinity jointly selected for different zoosporic parasites at the high and low ends of the salinity gradient.

We could not fully disentangle direct salinity constraints on parasitic chytrids from indirect salinity-mediated effects via host community. Variation partitioning showed that a majority of the variation explained by salinity and host diversity was shared between the predictors of interest and control variables (Table S6). The modest, unique effects of salinity and host diversity, together with a high proportion of unexplained variance in our model, underlines their role as contributing drivers among a complex range of other conditions, including additional unmeasured drivers and stochastic processes. Nonetheless, both salinity and host community composition remained significant predictors of parasitic chytrid communities along the salinity gradient, even after accounting for temporal structure and other measured covariates. The persistence of salinity and host-associated signals within this ecological complexity underscores their importance in structuring parasitic chytrid communities.

### Host–parasite infection dynamics

Overall, species-specific chytrid infection prevalence remained relatively low (Fig. 2B). In comparison, a previous study from the Baltic Sea reported prevalence of infection during a summer bloom to exceed 50% and reach up to 90% in host populations of *Oocystis* sp., *Monoraphidium contortum*, and *Chaetoceros minimus* populations [18]. Moreover, in temperate freshwater lakes, when conditions are favorable, infection prevalence during diatom spring blooms can reach up to 90% and is linked with a rapid decline of the infected host population [7, 73]. In contrast to these freshwater cases where high prevalence coincided with strong dominance by one or a few host species, the spring bloom in this study was more even and taxonomically mixed, which likely reduced the effective density of susceptible hosts and diluted chytrid transmission. It is worth noting, however, that even a low infection prevalence of zoosporic parasites may contribute to the decline of a host population and influence phytoplankton community dynamics [74].

Host–parasite dynamics between chytrids and their host taxa can vary interannually due to variation in both host population dynamics and abiotic conditions [73, 75, 76]. For example, temperature has been shown to influence the infection success of chytrid parasites of the freshwater diatom *A. formosa*, resulting in a temperature-mediated host refuge below 3°C [74]. During our sampling campaign, the biomass of host species *P. taeniata* and *Thalassiosira* sp. peaked at temperatures below 5°C (Figs 1B and 2). Consequently, such low temperatures could limit the performance of parasitic chytrids, resulting in temperature-mediated partial host refugia.

Several studies have shown a positive association between chytrid infectivity and temperature in phytoplankton populations [6, 8, 73, 77]. Along with climate change-induced shifts in temperatures, the impact of chytrids could become more pronounced in the Baltic Sea spring bloom. For example, warmer conditions might exert stress on cold-water-adapted diatoms, like *Thalassiosira* sp. and *P. taeniata*, making them more susceptible to infections, which could influence the outcome in competition with other taxa less affected by rising temperatures, for example, dinoflagellates [78]. On the other hand, as sea surface temperatures continue to rise, cold-water diatoms could possibly disappear from the spring bloom entirely [78, 79], and hence also associated parasites and their functions.

Alternatively, high genetic diversity within host populations has been hypothesized to hinder the spread of infection by chytrids [80, 81]. With high genetic diversity in the host population, genotype-level host specificity [8] could hamper infection rates due to lower density of true host populations and, consequently, lower parasite transmission. Based on the *rbcL* marker gene, there was genetic variability at the ASV level within the host populations of this study (Fig. S10), which could contribute to the low infection prevalence observed during the spring bloom.

We did not observe infections by chytrids on other abundant phytoplankton taxa, such as *Skeletonema marinoi*, *Chaetoceros* sp*.* or *Peridiniella catenata*, despite reports of chytrid infections on the genera *Skeletonema* and *Chaetoceros* in coastal environments [18, 82]. Mismatches in environmental tolerance between hosts and parasites [6, 73], absence of preferred genotypes in the host population [8], or chemical resistance against chytrid infections [83] could all be possible explanations behind the absence of infections in these phytoplankton species.

Consistent with the generally low detection of chytrid infections in the south, microscopy detected only a single infection event on *Thalassiosira* sp., whereas none were detected by IFCB. Because *Thalassiosira* was less abundant in the south, fewer images were analyzed by IFCB, thus a very low, sustained prevalence cannot be fully excluded. However, the combination of a single microscopy detection of infections on *Thalassiosira* sp. and otherwise repeated non-detection by IFCB argues that chytrid transmission was likely not sustained in the south. Molecular single-cell analysis showed that in the south, *Thalassiosira* sp. 2 and *G. delicatula* were infected by the same chytrid species, indicating a broader host range for this parasite. A generalist lifestyle is often associated with a trade-off between host range and growth rate [84, 85], which could disadvantage this chytrid in competition with specialist parasites of the dominant *G. delicatula*, consistent with the high infection prevalence of *Pirsonia* sp. and *Cryothecomonas* sp. on this host (Fig. 2). Thus, while parasitic chytrids adapted to local salinity and host conditions occur also in the southern Baltic Sea, infections likely failed to propagate due to low chytrid growth rates and competition with other zoosporic parasites.

Given that our study targeted eukaryotes using 18S and 28S rDNA markers (and *rbcl* for diatoms), we did not assess other microbial agents potentially influencing spring bloom dynamics, including viruses (e.g. through host lysis) and heterotrophic bacteria (e.g. via metabolic interactions and organic-matter recycling) [86–88]. Consequently, bacterial and viral contributions are not resolved here, limiting inference about the relative importance of parasitic chytrids in bloom progression or termination. Moreover, these compartments likely interact within a broader plankton interactome [89]. Future studies should therefore quantify bacterial and viral dynamics alongside chytrid parasites and use multi-omics [90, 91] and food web modeling approaches [92] to disentangle their relative and interacting roles in shaping phytoplankton bloom dynamics.

## Conclusion

We found that parasitic chytrids were driven by a complex interplay between salinity and host dynamics along the salinity gradient in the Baltic Sea. Both parasitic chytrid and their associated host communities were separated by a salinity threshold of 8, and a shift in phytoplankton host community composition was driving parasite community turnover along the salinity gradient. Our study suggests that the distribution and infection dynamics of chytrid parasites along the salinity gradient are shaped more by biotic interactions such as host availability, parasite competition, and co-adaptation than by the direct physiological effects of salinity itself. Controlled experiments on the salinity tolerance and performance of parasitic chytrids would help clarify whether their distribution is primarily limited by physiological constraints or mediated indirectly through host availability. In addition, we showed that chytrid communities were dominated by parasites during the phytoplankton bloom, highlighting their potential roles in structuring diversity and ecosystem functions. This study serves as a baseline for further research on the drivers of host–parasite associations between phytoplankton and parasitic chytrids in brackish and marine systems. Further research on phytoplankton-chytrid dynamics, combining molecular and quantitative data from year-round and annual sampling campaigns, together with experimental studies of environmental and biotic drivers, is needed to better understand how parasitic chytrid diversity contributes to phytoplankton and food web dynamics in marine and brackish environments.

## Supplementary Material

Supplementary_material_ycag269

## Data Availability

Raw amplicon sequence data are available in Sequence Read Archive (NCBI) under project number PRJNA1429088. Metadata and ASVs of parasitic chytrids can be found in Supplementary Data 1 and 2. Sanger sequences are available in GenBank (NCBI) with accession numbers provided in the Supplementary Data 3. Supplementary Methods and results are available in the Supplementary Information of this article.
