# Supplementary material for: Salinity and diatom host community jointly shape chytrid parasite diversity along a natural salinity gradient
Source: ISME Commun. 2026 Sep 25;6(1):ycag269. doi: 10.1093/ismeco/ycag269 (PMC13653609; doi:10.1093/ismeco/ycag269)
Supplement: Supplementary file 1 [file supplementary_material_ycag269.zip › Supplementary-Information_Kangas-et-al-ycag269.pdf]

Supplementary information: Salinity and diatom host community jointly shape chytrid parasite diversity along a natural salinity gradient

Jonna Kangas, Simon Bilik, Lumi Haraguchi, Kaisa Kraft, Maliheh Mehrshad, Conny Sjöqvist, Silke Van den Wyngaert

1. Supplementary methods

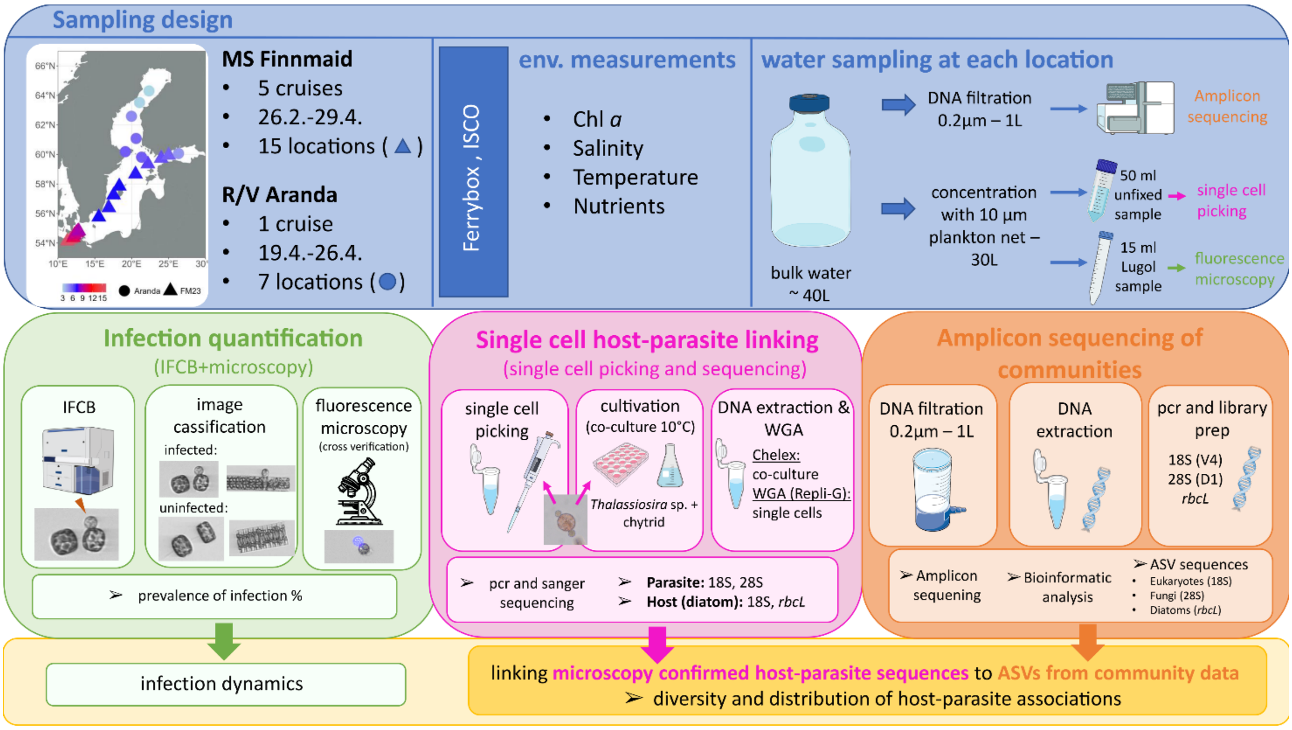

Figure S1. Schematic figure of the implemented workflow. Figure S1 was created using vector illustrations adapted from Bioicons (bioicons.com, under CC-BY 4.0 license) and NIH BioArt Source (bioart.niaid.nih.gov).

1.1 Quantification of phytoplankton and parasite infections

The IFCB data was classified according to Kraft and colleagues [1] but with an updated classifier version, into taxonomic classes of higher and lower taxonomic detail using a Convolutional Neural Network model with a Resnet-18 architecture, pre-trained with ImageNet and finetuned with Baltic Sea data. Image-specific biovolumes were calculated using the distance map method described in Moberg and Sosik [2].

Classified images were further sorted into anomalous and non-anomalous images using the anomaly detection algorithm described in [3]. The sorted images were then manually checked for false classifications and further sorted into the category: infected, if based on morphological characteristics and image quality the infection was likely a parasite, and non-infected, if the phytoplankton cells or chains were not clearly infected by a parasite. An anomaly was considered a chytrid infection, if the initial attachment or sporangia was clearly attached to the host cell. An anomaly was considered an infection by *Pirsonia* sp. or *Chryothecomonas* sp. based on morphological features described in Cattlett et al. [4]. Prevalence of infection was calculated and

converted into infected biomass by multiplying infection prevalence with the biomass of the species in question. Prevalence of infection was calculated as follows: *infected/(uninfected+infected)*. Parasite infection results obtained from the IFCB-anomaly detection method were cross-checked with microscopy. The samples were stained with two fungal cell wall markers, calcofluor white (CFW) and wheat germ agglutin (WGA) following Klawonn et al. [5] to visualize fungal parasites. One mL of concentrated sample was transferred to a 24-well plate and examined at 100x or 200x magnification using an inverted microscope (Invitrogen EVOS M7000, Thermo Fischer Scientific). In most cases, the entire well was screened to obtain a minimum count of 100 cells per phytoplankton taxa. Phytoplankton were identified to class, genus or species level and the number of infected and uninfected cells was counted to obtain cell abundances and prevalence of infection.

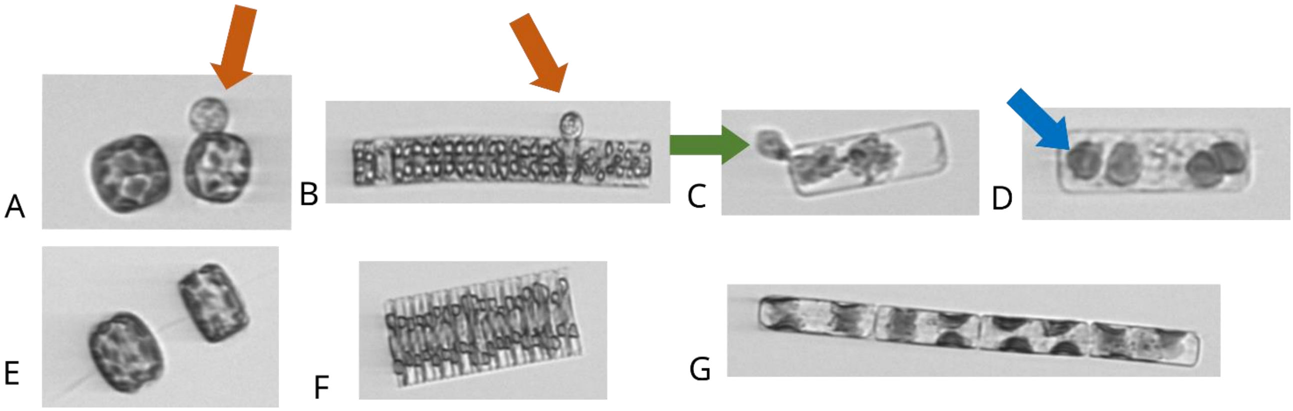

Figure S2. Examples of infected (anomalous) and uninfected (non-anomalous) phytoplankton cells from IFCB image data: A) infected *Thalassiosira* sp. chain, B) infected *Pauliella taeniata* chain, C-D) infected Guinardia delicatula chain, E) healthy *Thalassiosira* sp. chain, F) healthy *Pauliella taeniata* chain, G) healthy *Guinardia delicatula* chain. Orange arrows indicate putative chytrid infections, green arrow putative *Prisonia* sp. infection and blue arrow indicates putative *Cryothecomonas* sp. infection

1.2 Single cell isolation and cultivation, whole genome amplification and sequencing

PCR cycling conditions for individual primer sets:

18S region, NS1/NS4 [6]:
Initial denaturation at 94 C° for 2 minutes, 32 cycles of denaturation, annealing and elongation at 94 C° for 15 seconds, 53 C° for 15 seconds and 72 C° for 30 seconds, followed by final elongation for 10 minutes at 72 C°

28S region, LROR/LR5 [7, 8]:
Initial denaturation at 95 C° for 2 minutes, 32 cycles of denaturation, annealing and elongation at 95 C° for 20 seconds, 53 C° for 20 seconds and 72 C° for 40 seconds, followed by final elongation for 2 minutes at 72 C° for each primer pair respectively

SSU-F1289/SSU-R1772 [9]:

Initial denaturation at 94 C° for 2 minutes, 25 cycles of denaturation, annealing and elongation at 94 C° for 15 seconds, 56 C° for 30 seconds and 68 C° for 1 minute, followed by final elongation for 2 minutes at 68 C°.

rbcL-F [10, 11]:
Initial denaturation at 94 C° for 2 minutes, 45 cycles of denaturation, annealing and elongation at 94 C° for 1 minute, 56,3 C° for 45 seconds and 72 C° for 1,5 minutes, followed by final elongation for 10 minutes at 72 C° for each primer pair respectively.

1.4 Statistical analyses

Table S1. Sub-basins with corresponding sampling locations and salinity ranges

sub-basin	sampling locations	salinity
Gulf of Bothnia and Åland Sea (GOB+ÅS)	BO3a, US5Ba, F16a, F64a, SR7a, IU7a	3-5
Gulf of Finland (GOF)	15-13, LL3a	5-6
Baltic Proper and Bornholm Basin (BP+BB)	12-8	7
Arkona Basin and Bay of Mecklenburg (AB+BoM)	7-1	8-16

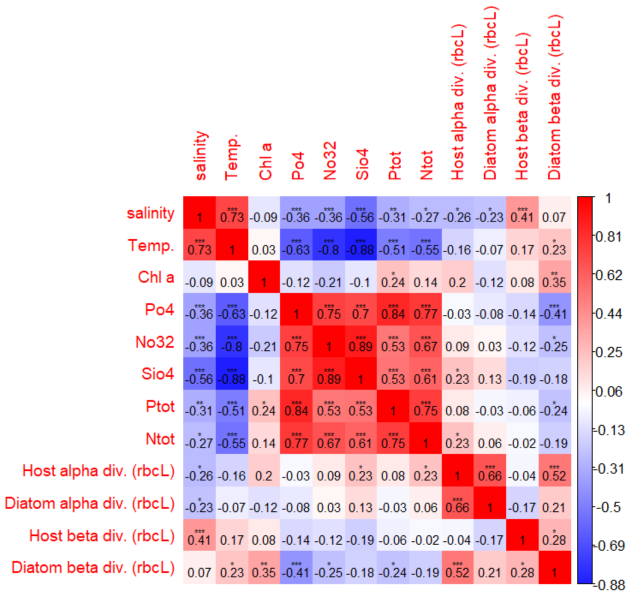

Figure S3. Correlation test for multicollinearity of predictor variables based on Spearman's rank correlation

2. Supplementary Results

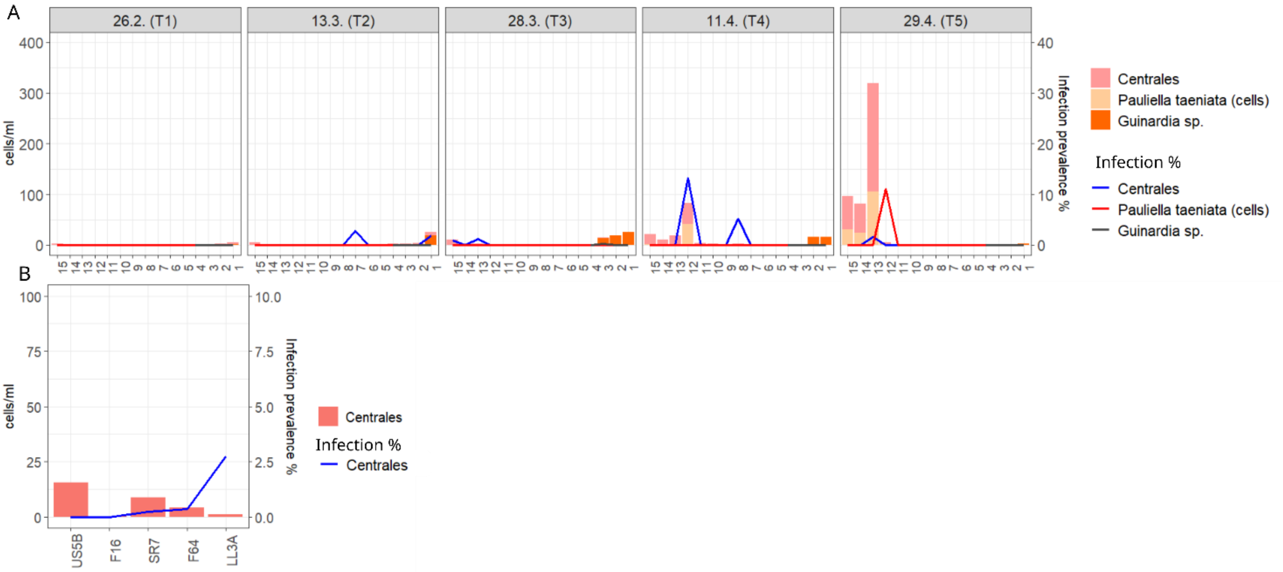

Figure S4. Microscopy data from lugol fixed samples. Cell abundance (cells/ml) of host species and infection prevalence % of A) Finnmaid transect, B) Aranda transect. In the northernmost locations in the Bothnian Bay (BO3a), there was still an ice cover with little biological activity.

A

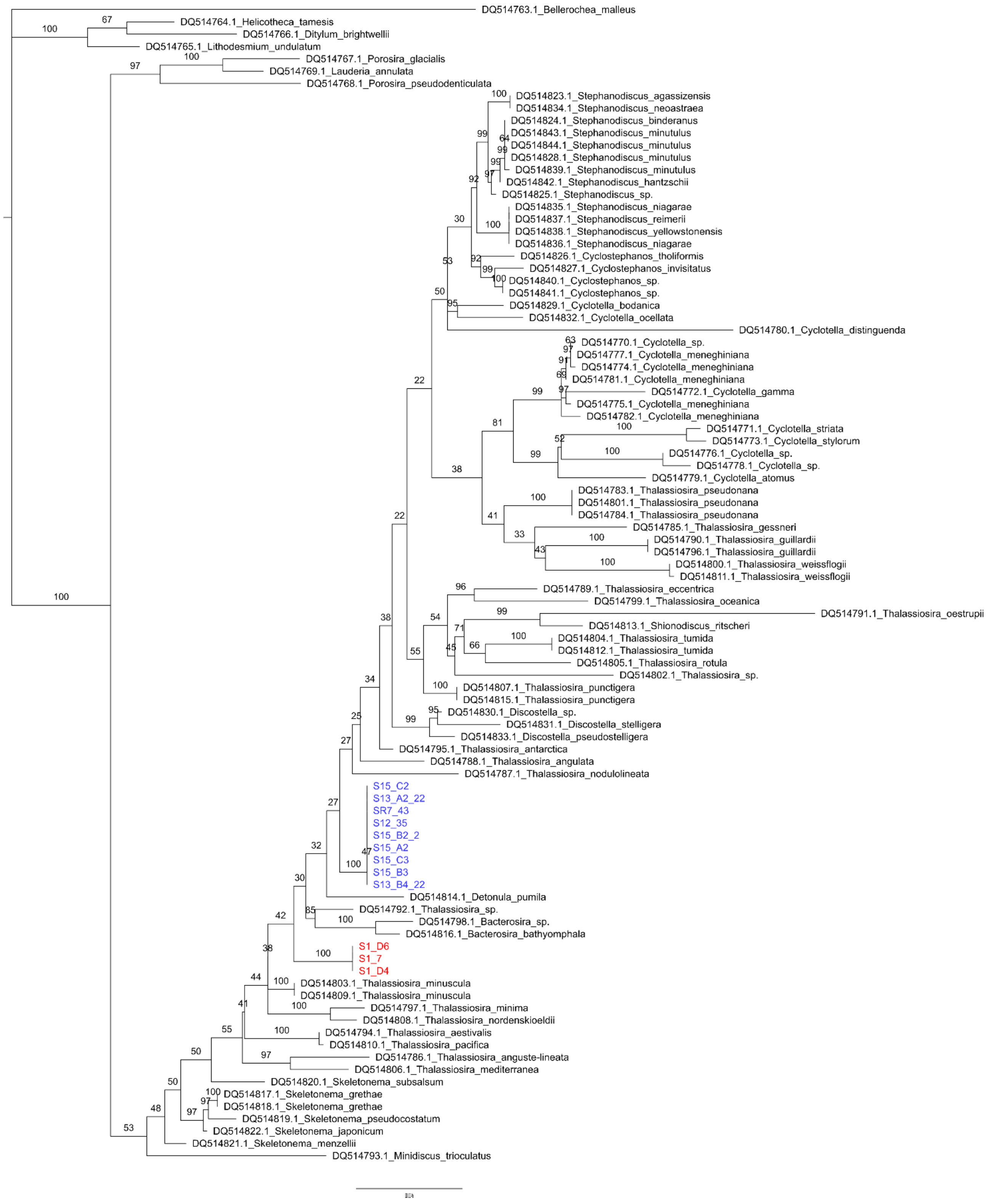
DQ514763.1_Bellerochea_malleus
DQ514764.1_Helicotheca_tamesis
DQ514766.1_Ditylum_brightwellii
DQ514765.1_Lithodesmium_undulatum
DQ514767.1_Porosira_glacialis
DQ514769.1_Lauderia_annulata
DQ514768.1_Porosira_pseudodenticulata
DQ514823.1_Stephanodiscus_agassizensis
DQ514834.1_Stephanodiscus_neoastraea
DQ514824.1_Stephanodiscus_binderanus
DQ514843.1_Stephanodiscus_minutulus
DQ514844.1_Stephanodiscus_minutulus
DQ514828.1_Stephanodiscus_minutulus
DQ514839.1_Stephanodiscus_minutulus
DQ514842.1_Stephanodiscus_hantzschii
DQ514825.1_Stephanodiscus_sp.
DQ514835.1_Stephanodiscus_niagarae
DQ514837.1_Stephanodiscus_reimerii
DQ514838.1_Stephanodiscus_yellowstonensis
DQ514836.1_Stephanodiscus_niagarae
DQ514826.1_Cyclostephanos_tholiformis
DQ514827.1_Cyclostephanos_invisitatus
DQ514840.1_Cyclostephanos_sp.
DQ514841.1_Cyclostephanos_sp.
DQ514829.1_Cyclotella_bodanica
DQ514832.1_Cyclotella_ocellata
DQ514780.1_Cyclotella_distinguenda
DQ514770.1_Cyclotella_sp.
DQ514777.1_Cyclotella_meneghiniana
DQ514774.1_Cyclotella_meneghiniana
DQ514781.1_Cyclotella_meneghiniana
DQ514772.1_Cyclotella_gamma
DQ514775.1_Cyclotella_meneghiniana
DQ514782.1_Cyclotella_meneghiniana
DQ514771.1_Cyclotella_striata
DQ514773.1_Cyclotella_stylorum
DQ514776.1_Cyclotella_sp.
DQ514778.1_Cyclotella_sp.
DQ514779.1_Cyclotella_atomus
DQ514783.1_Thalassiosira_pseudonana
DQ514801.1_Thalassiosira_pseudonana
DQ514784.1_Thalassiosira_pseudonana
DQ514785.1_Thalassiosira_gessneri
DQ514790.1_Thalassiosira_guillardii
DQ514796.1_Thalassiosira_guillardii
DQ514800.1_Thalassiosira_weissflogii
DQ514811.1_Thalassiosira_weissflogii
DQ514789.1_Thalassiosira_eccentrica
DQ514799.1_Thalassiosira_oceanica
DQ514791.1_Thalassiosira_oestrupii
DQ514813.1_Shionodiscus_ritscheri
DQ514804.1_Thalassiosira_tumida
DQ514812.1_Thalassiosira_tumida
DQ514805.1_Thalassiosira_rotula
DQ514802.1_Thalassiosira_sp.
DQ514807.1_Thalassiosira_punctigera
DQ514815.1_Thalassiosira_punctigera
DQ514830.1_Discostella_sp.
DQ514831.1_Discostella_stelligera
DQ514833.1_Discostella_pseudostelligera
DQ514795.1_Thalassiosira_antarctica
DQ514788.1_Thalassiosira_angulata
DQ514787.1_Thalassiosira_nodulolineata
S15_C2
S13_A2_22
SR7_43
S12_35
S15_B2_2
S15_A2
S15_C3
S15_B3
S13_B4_22
DQ514814.1_Detonula_pumila
DQ514792.1_Thalassiosira_sp.
DQ514798.1_Bacterosira_sp.
DQ514816.1_Bacterosira_bathyomphala
S1_D6
S1_7
S1_D4
DQ514803.1_Thalassiosira_minuscula
DQ514809.1_Thalassiosira_minuscula
DQ514797.1_Thalassiosira_minima
DQ514808.1_Thalassiosira_nordenskioeldii
DQ514794.1_Thalassiosira_aestivalis
DQ514810.1_Thalassiosira_pacifica
DQ514786.1_Thalassiosira_anguste-lineata
DQ514806.1_Thalassiosira_mediterranea
DQ514820.1_Skeletonema_subsalsum
DQ514817.1_Skeletonema_grethae
DQ514818.1_Skeletonema_grethae
DQ514819.1_Skeletonema_pseudocostatum
DQ514822.1_Skeletonema_japonicum
DQ514821.1_Skeletonema_menzellii
DQ514793.1_Minidiscus_trioculatus

B

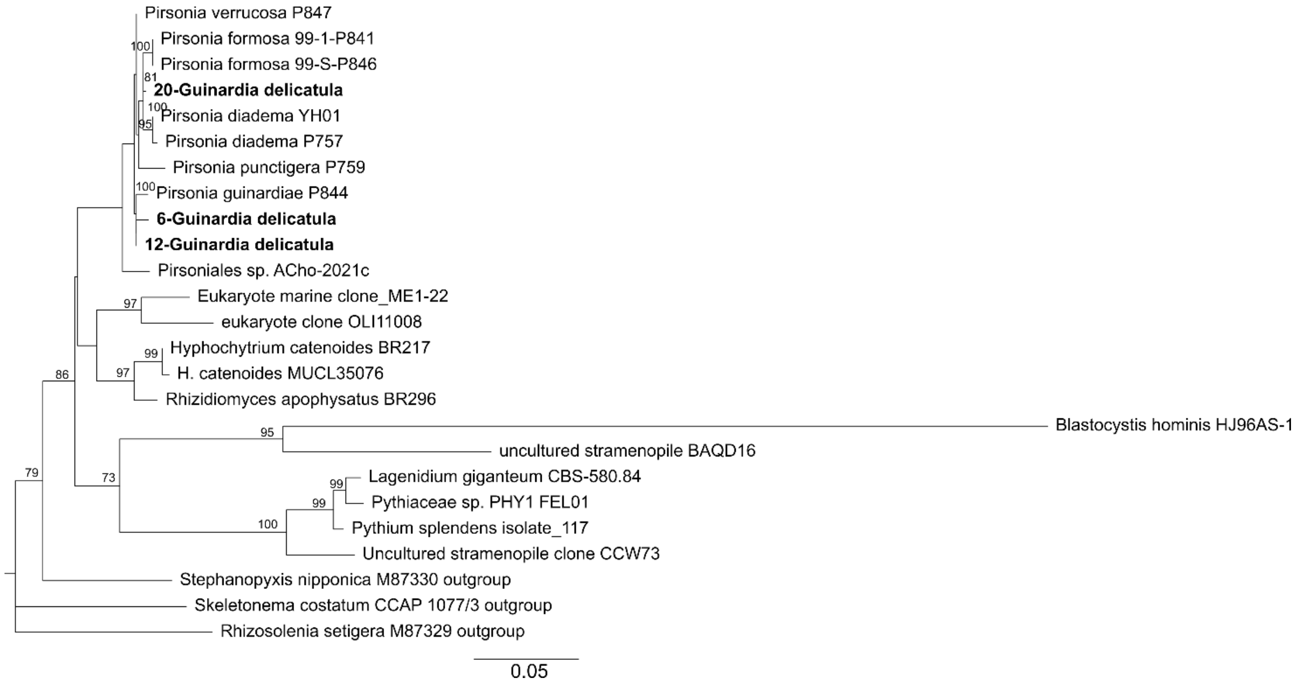

Figure S5. Phylogenetic tree of A) the *rbcL* region of the obtained **diatom host** sequences (700bp). Isolates marked in blue correspond to diatoms isolated from northern stations SR7, S15, S13 and S12 and isolates marked in red correspond to diatoms isolated from the southern station S1. B) the 18S region of parasitic stramenopile Pirsonia sp. identified on single cell samples of infected *Guinardia delicatula* cells, all isolated from the station S1 in the south (salinity 16).

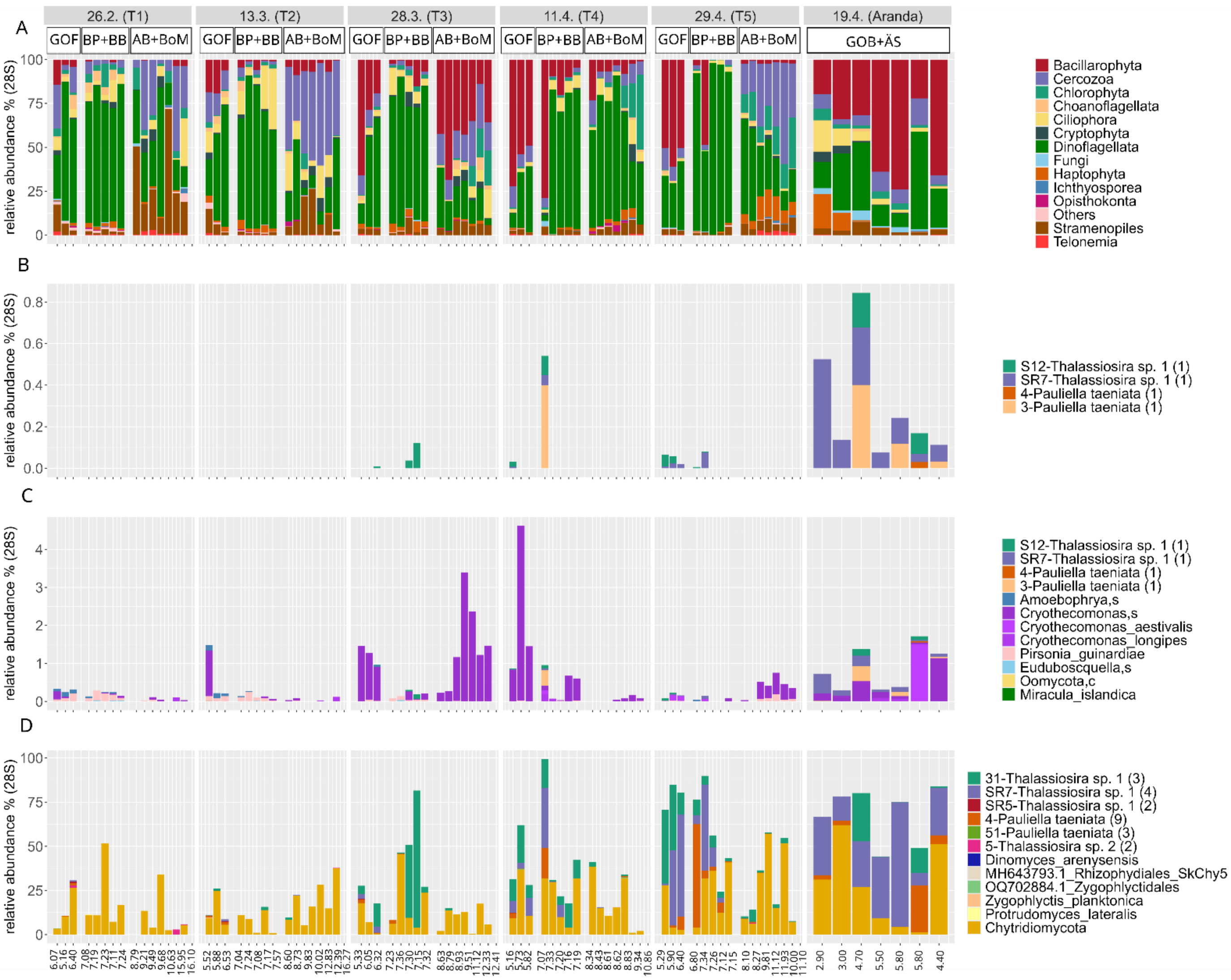

Figure S6 A) 18S eukaryotic community composition, B) 18S relative abundances of parasitic chytrid isolates from this study, C) 18S relative abundances of parasitic chytrid isolates from this study and other zoosporic parasites present in the ParaquaSeq database, D) 28S relative abundances of parasitic chytrid isolates from this study and present in the ParaquaSeq database.

Table S2 Relative abundance % of A) Chytridiomycota in the 28S Fungal communities, B) parasitic chytrids in the 28S Fungal communities at each sampling trip, C) host species in the diatom community. Values are given as mean ± standard deviation and the range of relative abundance in each sub-basin. *Note that samples for GoB + ÅS were collected between 19 and 26 April.

A				
**Trip**	**GoB + ÅS**	**GoF**	**BP + BB**	**AB + BoM**
26.2. (T1)		15 ± 14 (4-31)	20 ± 18 (7-52)	9 ± 12 (0-34)
13.3. (T2)		15 ± 10 (9-26)	7 ± 6 (1-16)	19 ± 11 (5-38)
28.3. (T3)		17 ± 10 (7-28)	43 ± 27 (8-82)	9 ± 7 (1-18)

11.4. (T4)	68 ± 16 (44-80)	40 ± 19 (28-62)	42 ± 33 (17-99)	17 ± 15 (10-41)
29.4. (T5)		78 ± 7 (71-85)	58 ± 26 (24-90)	28 ± 21 (8-58)
**B**				
**Trip**	**GoB + ÅS**	**GoF**	**BP + BB**	**AB + BoM**
26.2. (T1)		2 ± 2 (0-4)	0 ± 0 (0-0)	0 ± 1 (0-3)
13.3. (T2)		2 ± 1 (1-3)	0 ± 1 (0-2)	0 ± 1 (0-1)
28.3. (T3)		9 ± 8 (0-17)	25 ± 34 (1-77)	0 ± 0 (0-1)
11.4. (T4)	41 ± 17 (16-71)	21 ± 4 (22-24)	21 ± 27 (1-68)	0 ± 1 (0-3)
29.4. (T5)		67 ± 21 (21-67)	33 ± 30 (2-72)	2 ± 2 (0-7)
**C**				
**Trip**	**GoB + ÅS**	**GoF**	**BP + BB**	**AB + BoM**
26.2. (T1)		4 ± 2 (3-6)	1 ± 1 (0-14)	12 ± 19 (0-5)
13.3. (T2)		5 ± 3 (2-9)	0.4 ± 0.7 (0-1)	11 ± 22 (0-60)
28.3. (T3)		10 ± 6 (6-17)	0.4 ± 0.3 (0-0.7)	12 ± 16 (0-32)
11.4. (T4)	47 ± 17 (27-78)	16 ± 3 (13--17)	6 ± 6 (0.3-16)	19 ± 31 (0.1-66)
29.4. (T5		21 ± 3 (18-25)	26 ± 15 (2-45)	21 ± 3 (0.3-9)

113

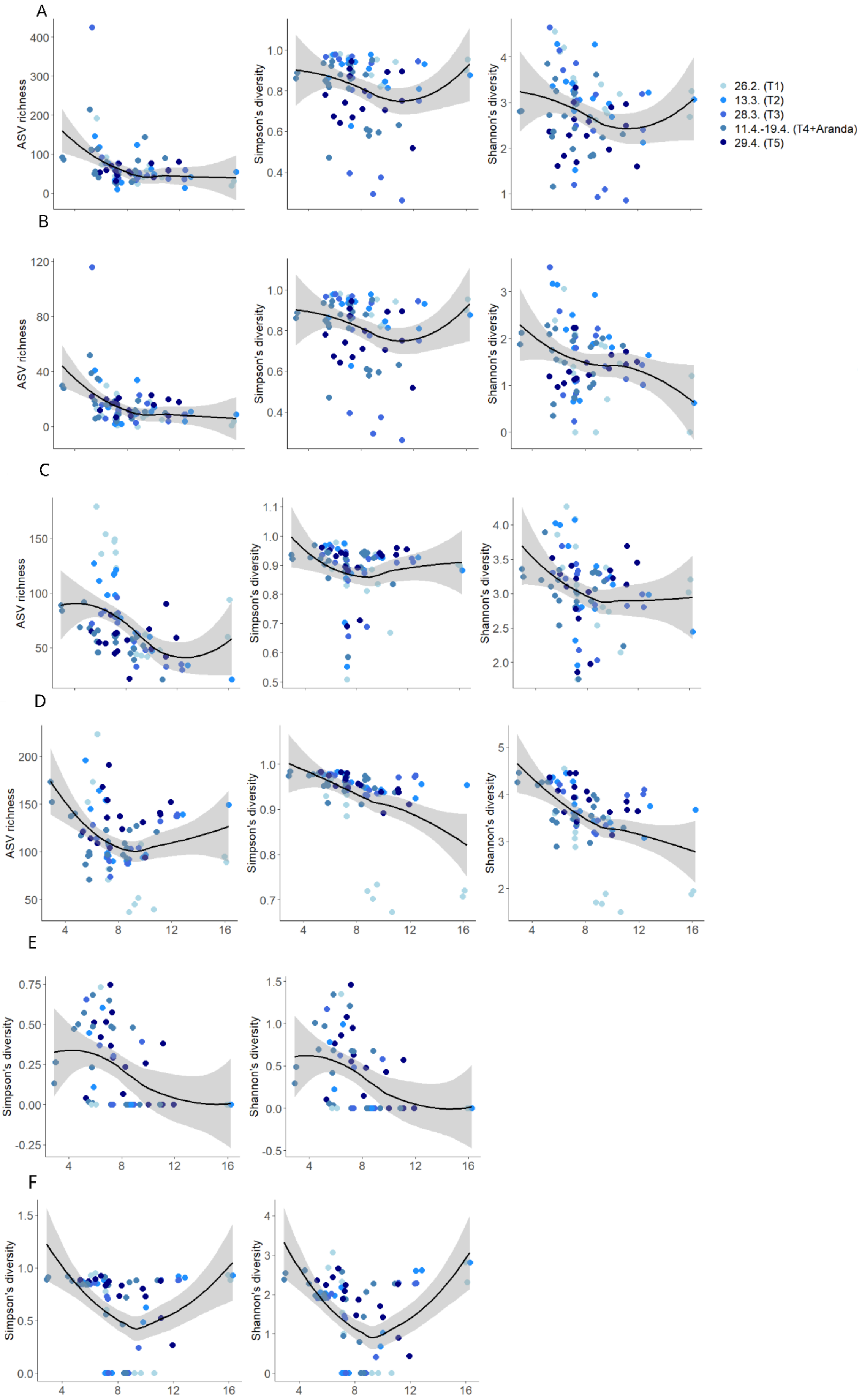
A
B
C
D
E
F
ASV richness
Simpson's diversity
Shannon's diversity
26.2. (T1)
13.3. (T2)
28.3. (T3)
11.4.-19.4. (T4+Aranda)
29.4. (T5)

Figure S7. Relationship of alpha diversity and salinity at different timepoints for A) Fungi (28S); B) Chytridiomycota (28S); C) Eukaryotes (18S); D) Bacillariophyta (rbcL); E) parasitic chytrids (28S); F) Host ASVs (rbcL).

Table S3. Post-hoc test using pairwise.adonis2 for testing pairwise differences in community composition between sub-basins. A) parasite communities, B) host communities

A					
**comparison**	**Df**	**SS**	**R2**	**F**	**p-value**
GOB+ÅS vs AB+BoM	1	2.275	0.233	7.272	0.001 ***
GOB+ÅS vs. GOF	1	0.7388	0.112	2.403	0.026 *
GOB+ÅS vs. BP+BB	1	1.143	0.169	4.066	0.003 **
AB+BoM vs. GOF	1	1.590	0.119	4.474	0.001 ***
AB+BoM vs. BP+BB	1	1.118	0.089	3.312	0.003 **
GOF vs. BP+BB	1	0.330	0.033	0.976	0.457
B					
**comparison**	**Df**	**SS**	**R2**	**F**	**p-value**
GOB+ÅS vs AB+BoM	1	2.754	0.180	7.923	0.001 ***
GOB+ÅS vs. GOF	1	0.100	0.273	7.515	0.002 **
GOB+ÅS vs. BP+BB	1	1.767	0.173	5.838	0.001 ***
AB+BoM vs. GOF	1	3.794	0.206	11.934	0.001 ***
AB+BoM vs. BP+BB	1	1.341	0.062	3.542	0.001 ***
GOF vs. BP+BB	1	1.447	0.120	5.194	0.004 **

Table S4. Post-hoc test using TukeysHSD for testing pairwise differences in homogeneity of dispersion between sub-basins. A) parasite communities, B) host communities

A			
comparison	mean difference	confidence intervals	p adjusted
GOB+ÅS vs. GOF	0.230	-0.016, 0.475	0.074
GOB+ÅS vs. BP+BB	-0.209	-0.452, 0.016	0.117
GOB+ÅS vs. AB+BoM	-0.221	-0.457, 0.183	0.076
GOF vs. BP+BB	0.021	-0.161, 0.204	0.990
GOF vs. AB+BoM	0.009	-0.164, 0.183	0.999
BP+BB vs. AB+BoM	-0.012	-0.182	0.159
B			
comparison	mean difference	confidence intervals	p adjusted
GOB+ÅS vs. GOF	0.157	-0.010	0.074
GOB+ÅS vs. BP+BB	-0.373	-0.533, -0.214	<0.001***

GOB+ÅS vs. AB+BoM	-0.418	-0.573, -0.263	<0.001***
GOF vs. BP+BB	-0.217	-0.329, -0.104	<0.001***
GOF vs. AB+BoM	-0.261	-0.367, -0.154	<0.001***
BP+BB vs. AB+BoM	-0.044	-0.138, 0.050	0.606

Table S5. Results from distance-based redundancy analysis testing for the unique contributions of salinity and diatom host diversity on parasitic community composition along the sampling transect (salinities 3-15).

**Eigenvalues:** CAP1: 1.235, CAP2: 0.658				
**Proportion explained:** CAP1: 0.596, CAP2: 0.913				
Model p: 0.001***				
	**Df**	**Sum of Squared**	**F**	**p-Value**
salinity	1	0.649	2.201	0.011*
Host alpha diversity (*rbcL*)	1	0.477	1.617	0.076
Host beta diversity (*rbcL*)	1	0.576	1.953	0.034*
Residual	46	13.553		
**Partitioning of squared Bray distance**				
	Inertia	Proportion		
Total	21.853	1.000		
Conditioned	6.226	0.285		
Constrained	2.074	0.095		
Unconstrained	13.553	0.620		

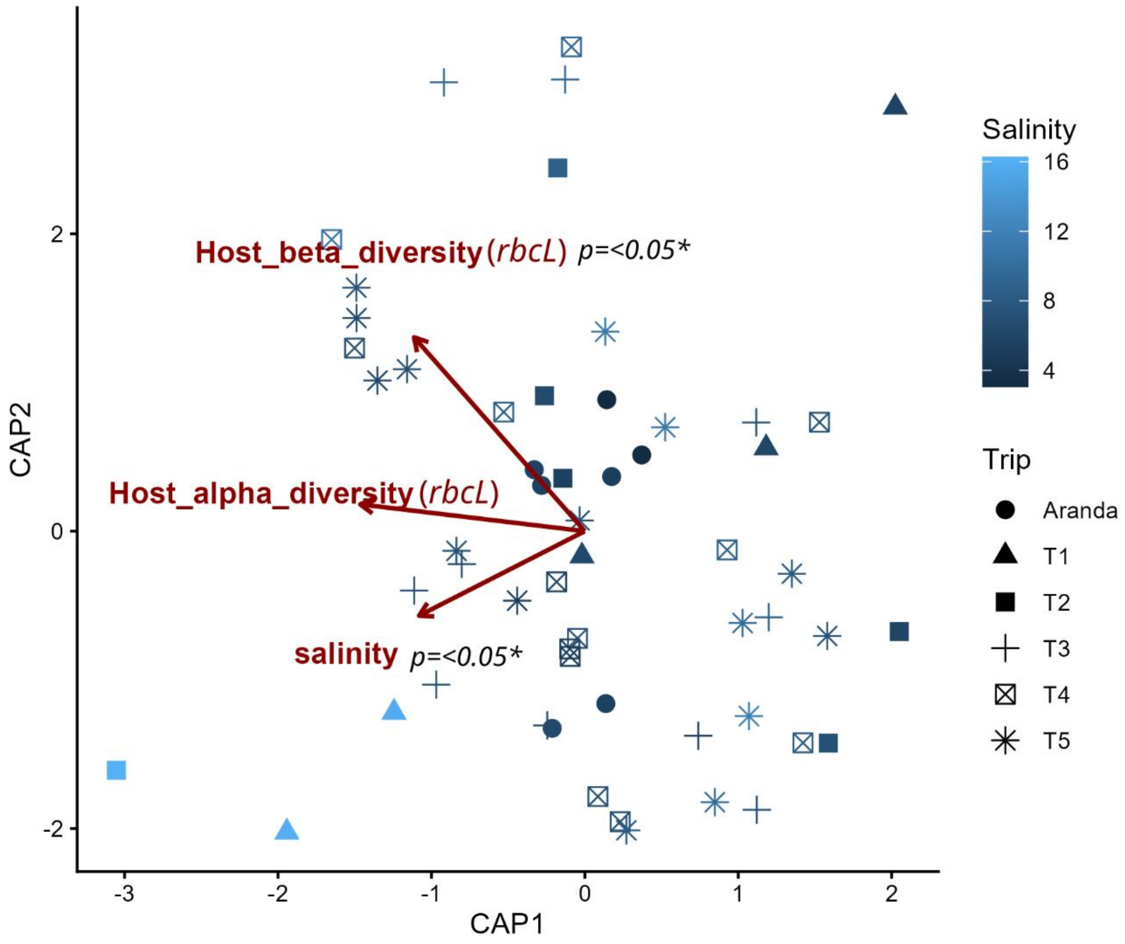

Figure S8. Distance-based redundancy analysis (dbRDA) ordination of parasitic chytrid community composition with vectors for salinity, host alpha diversity and host beta diversity (see Table S6 for model statistics).

Table S6. Variance partitioning of shared and unique contributions of salinity, host diversity including alpha and beta diversity, and control variables including sampling trip and temperature.

**Unique fractions and total with shared fractions equally allocated**		
	unique	contributed
salinity	0.021	0.048
host alpha and beta diversity	0.029	0.078
sampling trip and temp.	0.092	0.152
**Partition of squared Bray distance in dbRDA**		
	Df	Adj. R^2
unique contributions		
salinity	1	0.02076
host alpha and beta div.	2	0.02866
sampling trip and temp.	6	0.09205
shared contributions		
salinity + host alpha and beta div.	0	0.01666
salinity + sampling trip and temp.	0	0.03959
host div. + sampling trip and temp.	0	0.08249

Residuals		0.72

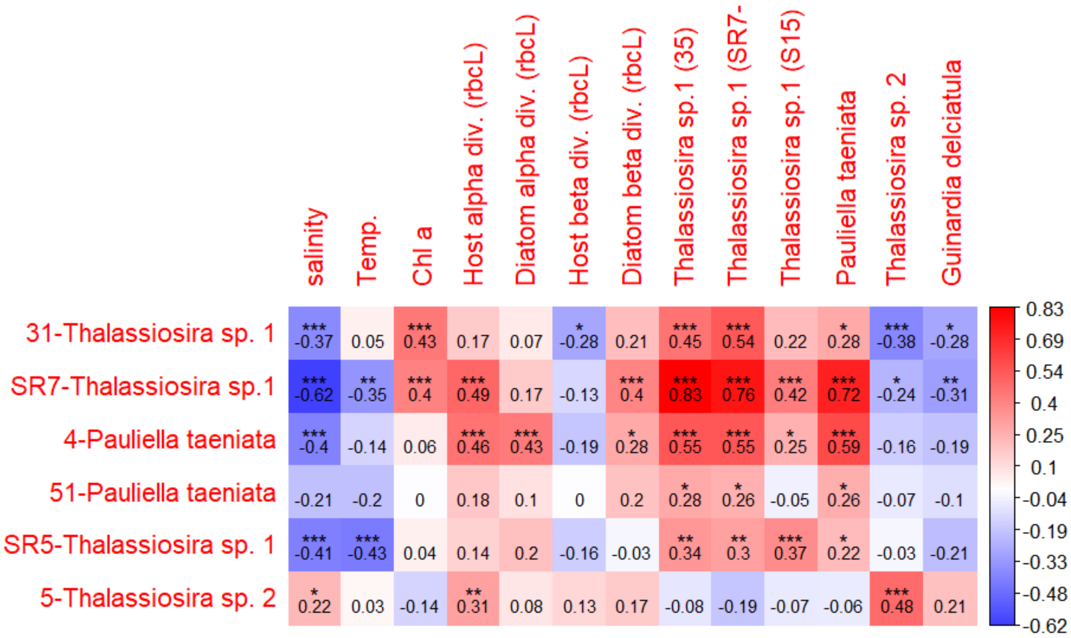

Figure S9. Spearman's correlation analysis of single cell parasitic chytrid ASVs against host ASVs, host and diatom community diversity metrics, salinity, temperature and Chl *a*

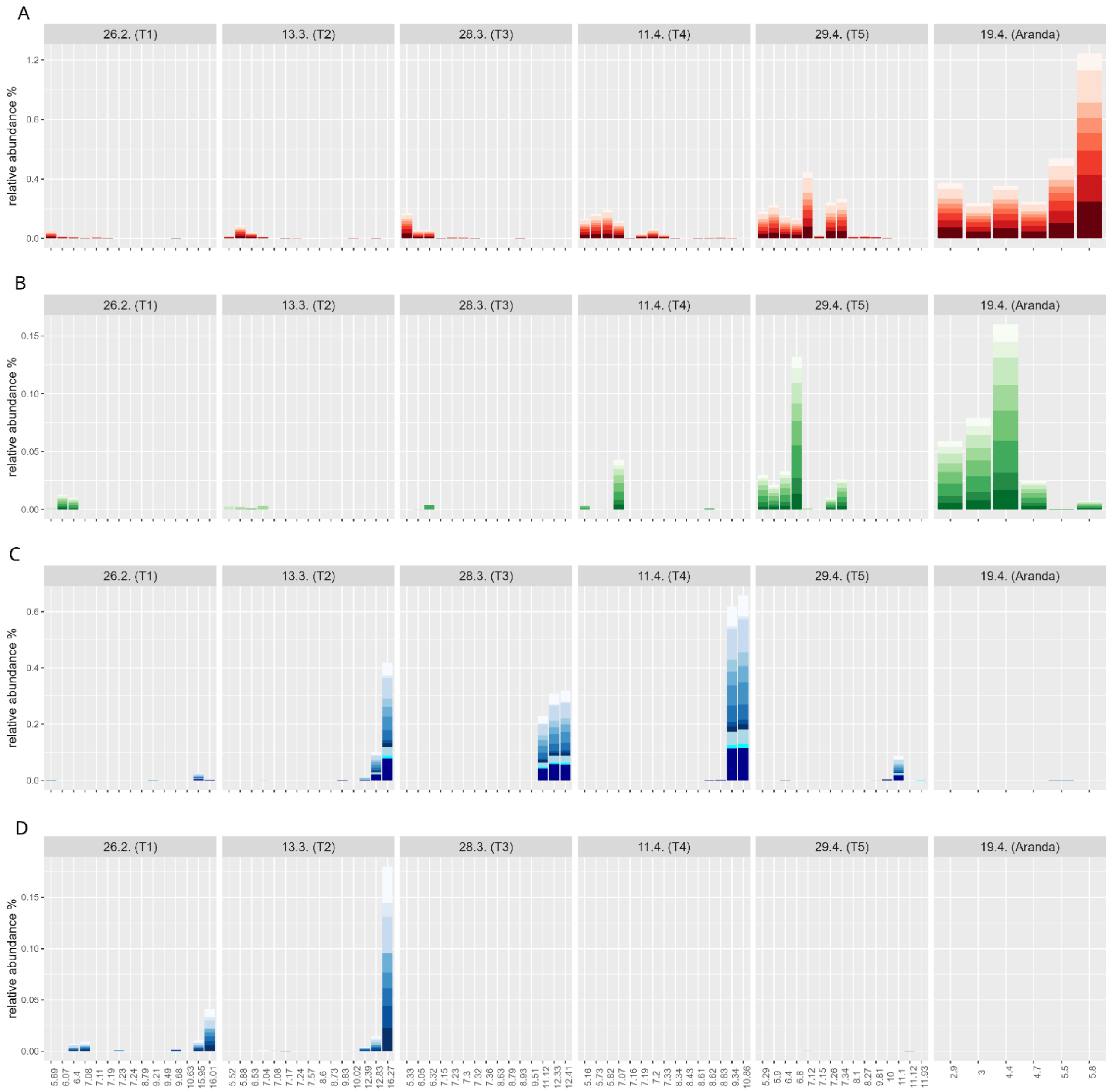

Figure S10. Genetic composition at ASV level based on rbcL data of Host species A) *Thalassiosira* sp.1; B) *Pauliella taeniata*; C) *Guinardia delicatula*; D) *Thalassiosira* sp.2.

References:

1. Kraft, K., Velhonoja, O., Eerola, T., Suikkanen, S., Tamminen, T., Haraguchi, L., ... & Seppälä, J. (2022). Towards operational phytoplankton recognition with automated high-throughput imaging, near-real-time data processing, and convolutional neural networks. Frontiers in Marine Science, 9, 867695.
2. Moberg, E. A., and Sosik, H. M. (2012). Distance maps to estimate cell volume from two-dimensional plankton images. Limnol. Oceanogr. Methods 10, 278–288. doi: 10.4319/lom.2012.10.278

3. Bilik, S., Batrakhanov, D., Eerola, T., Haraguchi, L., Kraft, K., Van den Wyngaert, S., ... & Horak, K. (2023). Toward phytoplankton parasite detection using autoencoders. Machine Vision and Applications, 34(6), 101.
4. Catlett, D., Peacock, E. E., Fontaine, D. N., Taylor Crockford, E., McKenzie, M. J., Rynearson, T. A., & Sosik, H. M. (2025). Concurrent DNA meta-barcoding and plankton imaging reveal novel parasitic infection and competition in a diatom. Limnology and Oceanography, 70, S86-S101.
5. Klawonn, I., Dunker, S., Kagami, M., Grossart, H. P., & Van den Wyngaert, S. (2023). Intercomparison of two fluorescent dyes to visualize parasitic fungi (Chytridiomycota) on phytoplankton. Microbial ecology, 85(1), 9-23.
6. White, T. J., Bruns, T., Lee, S. J. W. T., & Taylor, J. (1990). Amplification and direct sequencing of fungal ribosomal RNA genes for phylogenetics. PCR protocols: a guide to methods and applications, 18(1), 315-322.
7. Rehner, S. A., & Samuels, G. J. (1994). Taxonomy and phylogeny of Gliocladium analysed from nuclear large subunit ribosomal DNA sequences. Mycological Research, 98(6), 625-634.
8. Vilgalys R, Hester M. Rapid genetic identification and mapping of enzymatically amplified ribosomal DNA from several Cryptococcus species. J Bacteriol. 1990;172:4238–46.
9. Tanabe, A. S., Nagai, S., Hida, K., Yasuike, M., Fujiwara, A., Nakamura, Y., ... & Katakura, S. (2016). Comparative study of the validity of three regions of the 18S-rRNA gene for massively parallel sequencing-based monitoring of the planktonic eukaryote community. Molecular ecology resources, 16(2), 402-414.
10. Abarca, N., Jahn, R., Zimmermann, J., & Enke, N. (2014). Does the cosmopolitan diatom Gomphonema parvulum (Kützing) Kützing have a biogeography?. PLoS One, 9(1), e86885.
11. Turk Dermastia, T., Vascotto, I., Francé, J., Stanković, D., & Mozetič, P. (2023). Evaluation of the rbcL marker for metabarcoding of marine diatoms and inference of population structure of selected genera. Frontiers in Microbiology, 14, 1071379.
